# Double negative T cells decline in inflammatory reproductive dysfunction

**DOI:** 10.1038/s41598-026-57959-4

**Published:** 2026-06-21

**Authors:** Enitome E. Bafor, Toni Martin, Bruna Karoline Tatematsu, Ian A. Bettencourt, Adrienne E. Kimmel, Dorothy K. Sojka, Howard A. Young

**Affiliations:** 1 Cancer Innovation Laboratory, Center for Cancer Research, National Cancer Institute, Frederick, MD 21702 USA; 2 Microbiology and Immunology Department, Loyola University Health Science Campus, Maywood, IL 60153 USA; 3 Present Address: Department of Infectious Diseases and Immunology, College of Veterinary Medicine, University of Florida, Gainesville, Fl 32608 USA

**Keywords:** Double-negative T cells, CD8^+^ T cells, IFN-γ-mediated inflammation, Ovarian immune tolerance, Uterine immune tolerance, Diseases, Immunology

## Abstract

Double-negative T cells (DNTs; CD3^+^CD4^−^CD8^−^) have been implicated in immune regulation in autoimmune settings, but their relevance to reproductive tissue immune balance and inflammation-associated infertility remains unclear. Here, we define a population of TCRβ^+^NK1.1^−^ DNTs enriched in the mouse ovary and uterus and profiled its phenotype, tissue behavior, and function in inflammatory models associated with impaired fertility. In parallel, we performed RNA-seq on spleen- and thymus-derived NK1.1^−^ DNT and CD8^+^ T cell enriched population. Relative to CD8^+^ T cells, peripheral NK1.1^−^ DN T-cell-enriched populations displayed an activated, regulatory-like transcriptional profile with reduced *Cd8a*/*Cd8b1*, *Il7r*, and cytotoxic effector markers, alongside increased expression of *Pdcd1*, *Lag3*, *Tox*, and *Il10*. Ex vivo, FACS-sorted splenic DNTs produced low inflammatory cytokine output after CD3/CD28 stimulation and suppressed CD8^+^ T-cell proliferation primarily through contact-dependent mechanisms. In vivo, ovarian DNTs decreased following CD8-targeting depletion, and ovarian and uterine DNTs showed limited exchange in parabiosis. In chronic interferon-γ-driven inflammation (ARE^−/−^) and zona pellucida 3-induced ovarian inflammation, reproductive tissues exhibited reduced DNT frequencies, a shift in the DNT: CD8^+^T ratio favoring CD8^+^ T cells and increased activated CD8^+^ phenotypes. Finally, adoptive transfer of ex vivo FACS-sorted wild-type DNTs into ARE^−/−^ females increased pregnancy frequency, supporting DNT-associated immunoregulation as a feature of inflammation-associated infertility.

**Supplementary Information:**

The online version contains supplementary material available at https://doi.org/10.1038/s41598-026-57959-4.

## Introduction

Immune and inflammatory disorders are major, often under-recognized drivers of female reproductive dysfunction. Autoimmune diseases disproportionately affect women and many of these conditions have direct reproductive consequences^1,2^. In parallel, inflammatory gynecologic conditions such as endometriosis and adenomyosis are common in patients undergoing infertility evaluation or treatment^2^. Across infertility phenotypes, immune mechanisms have been implicated in unexplained infertility, recurrent implantation failure, pregnancy complications, and premature loss of ovarian function^3–5^. Clinical and mechanistic studies link altered T-cell composition, Th1/Th17-skewed cytokine programs, and local immune activation in reproductive tissues to impaired follicular development, embryo implantation, and placentation in inflammatory disease settings^5–7^.

Experimental models have been essential for dissecting these pathways. Immunization with zona pellucida 3 (ZP3) peptides induces T-cell-driven autoimmune oophoritis, follicle depletion, and infertility^8,9^. Mice with AU-rich element (ARE) deletion in the interferon-γ (IFN-γ) 3′ untranslated region (ARE^−/−^) develop chronically elevated IFN-γ, systemic autoimmunity, and reproductive dysfunction driven in part by aberrant CD8^+^ T-cell accumulation in reproductive tissues^10,11^. These clinical and experimental data support a model in which Th1-skewed and cytotoxic T-cell responses contribute to inflammation-associated reproductive dysfunction, while the local mechanisms that restrain these responses remain incompletely defined. A central question is how reproductive tissues constrain inflammatory and cytotoxic T-cell activity while still permitting host defense and physiologic tissue remodeling.

Regulatory T cells (Tregs) are relevant for implantation and pregnancy maintenance, and quantitative or functional defects in Tregs are linked to infertility and pregnancy loss^12,13^. Uterine and decidual Treg populations expand in response to conceptus and hormonal cues, and experimental disruption of Treg pathways can trigger implantation failure, fetal loss, or placental pathology^13–15^. Beyond CD4^+^Foxp3^+^Tregs, other non-conventional T cell subsets, including γδ T cells^16^ and double-negative (DNTs; CD3^+^CD4^−^CD8^−^) T cells^17^, have been implicated in tissue homeostasis and immune regulation.

DNTs are increasingly recognized as multifunctional regulators in autoimmunity, transplantation, and cancer^18–20^, where TCRαβ^+^CD3^+^CD4^−^CD8^−^populations can suppress effector T cells and modulate antigen-presenting cell function. Given that CD8^+^ T-cells are prominent effectors in autoimmune and IFN-γ-driven reproductive pathology^10,21–23^, understanding how local regulatory populations, including DNTs, interface with CD8^+^ T-cell responses is particularly important.

However, the phenotype, transcriptional program, and distribution of DNTs across nonpregnant reproductive tissues have not been systematically defined, and their behavior in inflammation-driven reproductive pathology remains unclear. This gap is compounded by DNT heterogeneity and multiple proposed developmental and peripheral routes for TCRαβ DNT subsets^24–29^.

To address these gaps, we defined how NK1.1^−^ TCRβ^+^ DNTs are distributed and programmed across female tissues at steady state and how this compartment changes in inflammatory conditions linked to reproductive dysfunction. Using multiparameter flow cytometry and RNA sequencing, we identify a reproductive tissue-enriched DNT compartment and show that splenic and thymic DNTs occupy an immunophenotypic and transcriptional state distinct from CD8^+^ T cells. In addition, we integrate suppression assays and cytokine profiling to define functional features consistent with a regulatory-biased program. We then assess tissue behavior using parabiosis and depletion approaches and test disease relevance across IFN-γ-driven inflammation (ARE^−/−^) and ZP3-CFA ovarian inflammation, including whether augmenting the DNT compartment by adoptive transfer modifies reproductive outcomes.

## Results

### Peripheral double-negative T cells are enriched in the ovary and uterus and display an activated phenotype

**Fig. 1:**
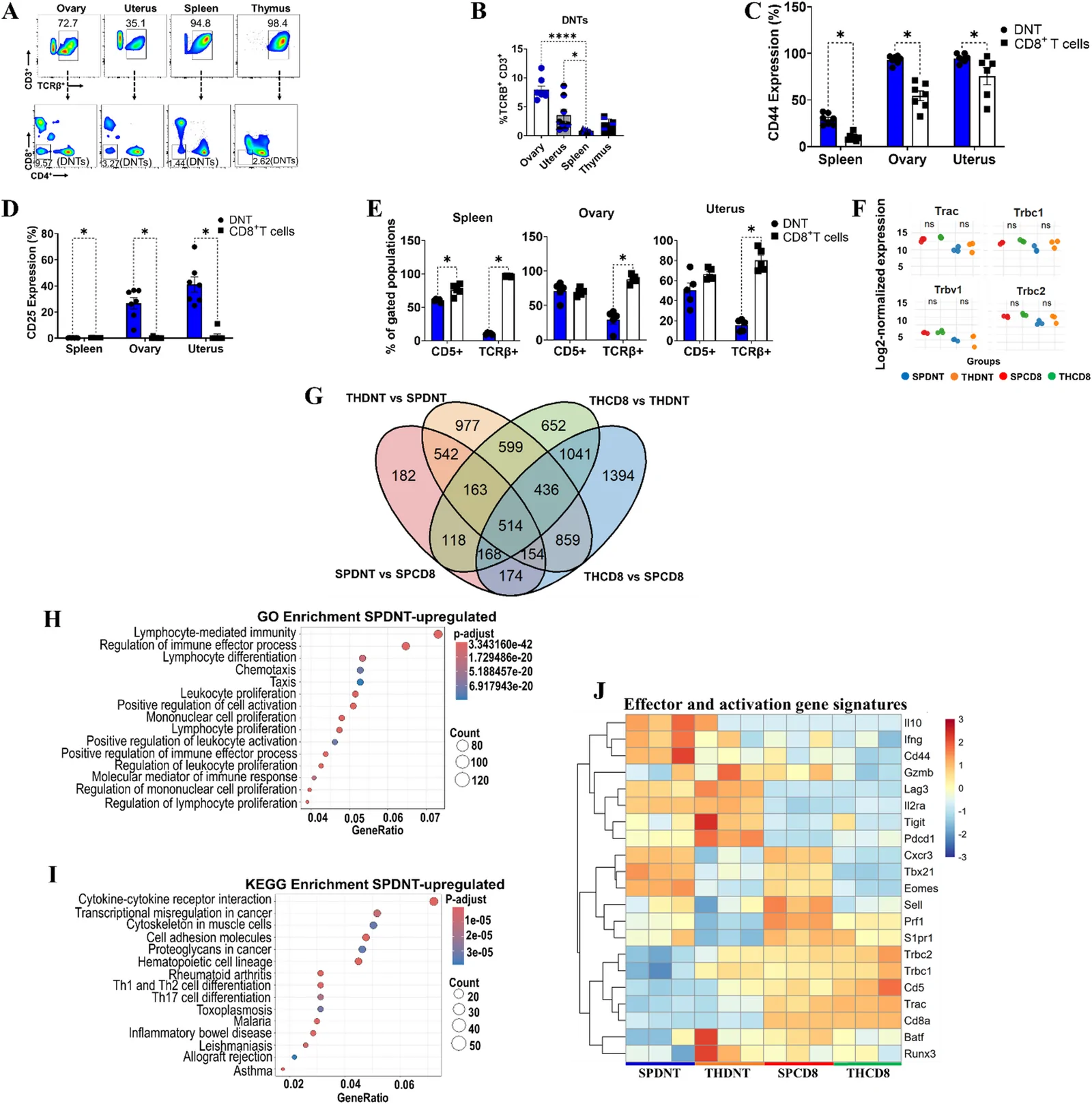
TCRβ^+^NK1.1^−^DNTs are enriched in the ovary and uterus, and NK1.1^−^DNT-enriched RNA-seq populations are transcriptionally distinct from CD8^+^ T cells. (**A**) Representative flow cytometry plots showing DNTs (CD3^+^TCRβ^+^CD4^−^CD8^−^) in the ovary, uterus, spleen, and thymus. Top row, CD3 versus TCRβ gating within live CD45^+^ singlets. Bottom row, CD4 versus CD8 plots of CD3^+^TCRβ^+^ cells with DNTs gated as CD4^−^CD8^−^. DNTs were identified within the live CD45^+^CD3^+^TCRβ^+^ gate and defined as CD4⁻CD8α⁻NK1.1⁻ cells (Full representative gating is shown in Fig. S2). Percentages indicate the frequency of DNTs among CD3^+^TCRβ^+^ cells in each tissue. (**B**) Frequency of DNTs among CD3^+^TCRβ^+^ T cells in ovary, uterus, spleen, and thymus (*n* = 4–8 mice per group, Kruskal-Wallis test). (**C**, **D**) Frequency of CD44^+^ (C) and CD25^+^ (**D**) cells within DNTs (blue-filled bars) and CD8^+^ T cells (open bars) from spleen, ovary, and uterus (*n* = 6–7 mice per group, Mann-Whitney test). (**E**) Frequency of CD5^+^ and TCRβ^+^ cells among gated DNTs and CD8^+^ T cells in spleen, ovary, and uterus. (**F**) Log_2_-normalized RNA-seq expression of TCR constant and variable region genes (*Trac*, *Trbc1*, *Trbc2*, *Trbv1*) in splenic DNTs (SPDNT), thymic DNTs (THDNT), splenic CD8^+^ T cells (SPCD8), and thymic CD8^+^ T cells (THCD8); ns, not significant. (**G**) Venn diagram showing overlap among direction-specific differentially expressed gene (DEG) sets across the indicated pairwise comparisons, illustrating shared and subset-specific transcriptional differences between tissues and lineages. For each comparison, the first-named population is compared relative to the second. (**H**, **I**) Gene Ontology (GO) biological process (**H**) and KEGG pathway^70,71^(**I**) enrichment analyses for genes upregulated in SPDNT relative to SPCD8. Dot size indicates the number of genes in each term and color indicates adjusted p-value. (**J**) Heat map of effector and activation gene signatures across SPDNT, THDNT, SPCD8, and THCD8 populations. Expression values are row-scaled (z-score); Corresponding log2 fold changes and adjusted p-values for highlighted genes are provided in Tables S6-S8. Statistical comparisons were performed using Mann Whitney tests. In all bar plots, data are shown as mean ± SEM; *p < 0.05, ****p < 0.0001; ns, not significant.

To define the steady-state distribution and activation-associated phenotype of peripheral double-negative T cells (DNTs) in WT females (prior to immunization or breeding), we compared DNT frequency and marker expression across reproductive tissues (ovary and uterus) and lymphoid tissues (spleen and thymus). Using flow cytometry, we consistently identified a discrete DNT population in all tissues examined on live, singlet CD45^+^ gated on CD3^+^TCRβ^+^ T cells (Fig. 1A and Fig. S1A). NK1.1⁺ cells were excluded (Fig. S1A), thereby defining a conventional NK1.1^−^ αβ DNT subset.

Despite smaller overall T-cell compartments in reproductive tissues, DNTs constituted approximately 8% of CD3⁺TCRβ⁺ T cells in the ovary, 4% in the uterus, 1% in the spleen, and 2.3% in the thymus (Fig. 1B), indicating relative enrichment in reproductive tissues compared with lymphoid organs. We next compared peripheral and thymic DNT phenotypes to assess evidence of antigen experience. Across spleen, ovary, and uterus, DNTs had higher CD44^+^ frequencies than tissue-matched CD8^+^ T-cells (Fig. 1C and Fig. S1B), consistent with an antigen-experienced phenotype^30,31^. Splenic DNTs (SPDNT) were largely CD25^−^, whereas ovarian and uterine DNTs showed higher proportions of CD25^+^ cells, approximately 30% and 40%, respectively, and these CD25^+^ fractions were increased relative to tissue-matched CD8^+^ T-cells (Fig. 1D and Fig. S1C). In contrast, thymic DNTs (THDNT) displayed comparatively low CD44 and CD25 relative to non-thymic DNTs (Fig. S1B-E), supporting phenotypic separation between thymic and peripheral DNT compartments.

Assessment of TCRβ and CD5 expression further distinguished peripheral from thymic DNTs. THDNTs expressed TCRβ and CD5 at levels comparable to thymic CD8^+^ T-cells (Fig. S1F-H). However, peripheral DNTs retained high CD5 expression with moderately reduced TCRβ relative to tissue-matched CD8^+^ T-cells (Fig. 1E and Fig. S1I-J), indicative of altered TCR expression and peripheral tuning^32^.

Prior studies indicate that TCRαβ^+^ DNT compartments are transcriptionally heterogeneous and can include subsets with cytotoxic or effector-associated programs that partially overlap with conventional T cell states, including CD8-associated differentiation modules^33,34^; therefore, we assessed canonical regulators of these programs (Runx3 and T-bet)^35,36^. Runx3 was highly expressed in double-positive thymocytes and detected at lower frequencies among DNTs and CD8^+^ T-cells across thymus and peripheral tissues (Fig. S1K-L). In addition, T-bet expression varied by tissue with SPDNT expressing higher T-bet than splenic CD8^+^ T cells, whereas uterine CD8^+^ T-cells expressed higher T-bet than uterine DNTs; in the ovary, DNTs and CD8^+^ T cells expressed similar T-bet levels (Fig. S1M). Together, these patterns suggest that DNTs share select transcriptional features with conventional CD8^+^ T-cells, while effector-associated programs are further tuned by the local tissue environment.

Because the RNA-seq sort used a reduced surface panel and did not include direct TCRβ or TCRγδ discrimination, these transcriptomic data were interpreted cautiously as representing an NK1.1⁻ DN T-cell-enriched population rather than a formally purified αβ DNT subset. Within this dataset, DN-enriched samples expressed conventional T-cell receptor αβ-associated transcripts including *Trac*, *Trbc1/2*, and representative *Trav* and *Trbv* genes at levels comparable to tissue-matched CD8^+^ T cells (Fig. 1F). We use these data to describe broader transcriptional features of the DN-enriched population with appropriate caution. Intersection analysis of differentially expressed genes from DNTs versus CD8^+^ T-cell comparisons revealed a shared core program alongside gene sets unique to each subset (Fig. 1G). Gene Ontology (GO) and Kyoto Encyclopedia of Genes and Genomes (KEGG) analyses of genes upregulated in SPDNT relative to SPCD8 showed enrichment for cytokine-cytokine receptor interactions and differentiation-associated pathways (Fig. 1H-I; Tables S2-S3). Conversely, genes downregulated in SPDNT, corresponding to programs elevated in SPCD8, were enriched for oxidative phosphorylation, ribosome biogenesis, and translation-related pathways (Fig. S1N-O; Tables S4-S5). In addition, DNTs were transcriptionally distinct from tissue-matched CD8⁺ T-cells (Fig. 1J; Table S6), suggestive of tissue-adapted activation and signaling programs. Detailed gating strategies are shown in Fig. S2.

Together, these data show that analytically defined TCRβ^+^NK1.1^−^ DNTs are enriched in the ovary and uterus and exhibit an activated, tissue-tuned phenotype, while bulk RNA-seq of a spleen- and thymus-derived NK1.1^−^ DN-enriched population identifies transcriptional features that distinguish that compartment from conventional CD8^+^ T cells.

### Peripheral DNTs suppress CD8^+^ T-cell proliferation through a primarily contact-dependent mechanism

**Fig. 2:**
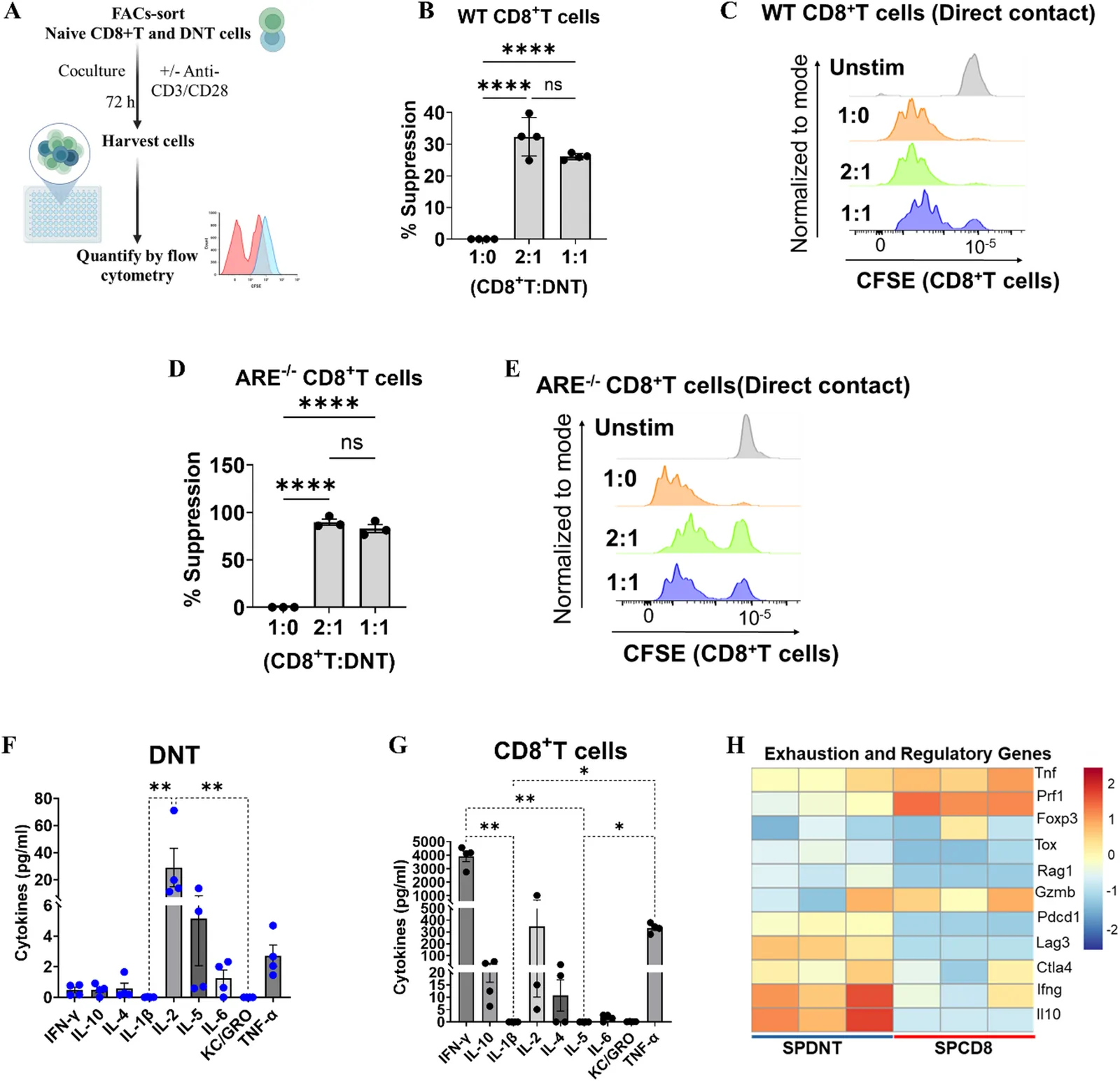
Peripheral DNTs suppress CD8^+^ T-cell proliferation through a primarily contact-dependent mechanism and exhibit a regulatory/exhaustion biased program. (**A**) Schematic of the CD8^+^ T-cell suppression assay. Splenic DNTs and splenic CD8^+^ T cells were FACS-sorted. CFSE-labeled CD8^+^ T cells were stimulated with anti-CD3/CD28 and cultured alone (1:0) or with DNTs at the indicated CD8^+^ T cell: DNT ratios for 72 h. CD8^+^ T-cell proliferation was quantified by CFSE dilution and percent divided by flow cytometry, and suppression was expressed as percent suppression relative to stimulated CD8^+^ T cells cultured without DNTs (1:0). This assay used polyclonal anti-CD3/CD28 stimulation and therefore assesses generalized suppression of activated CD8⁺ T-cell proliferation and not antigen-specific suppression. (**B**, **C**) Suppression of WT splenic CD8^+^ T-cell proliferation by splenic DNTs in direct-contact coculture (*n* = 4 biological replicates). (**B**) Percent suppression at the indicated CD8^+^ T cell: DNT ratios. (**C**) Representative CFSE histograms of proliferating CD8^+^ T cells under the same conditions (Unstim, unstimulated CD8^+^ T cells). (**D**, **E**) As in (**B**, **C**) but using CD8^+^ T cells isolated from ARE^−/−^ mice cocultured with WT splenic DNTs in direct contact (*n* = 3 biological replicates). (**D**) Percent suppression, (**E**) Representative CFSE histograms. (**F**, **G**) Cytokine production by DNTs (**F**) and CD8^+^ T cells (**G**) following 72 h stimulation with anti-CD3/CD28. Supernatants were assayed using multiplex electrochemiluminescence, and cytokine concentrations are presented as pg/mL (*n* = 4 biological replicates). (**H**) Heatmap of RNA-seq expression of selected regulatory, effector, and exhaustion-associated genes in splenic DNTs (SPDNT) and splenic CD8^+^ T cells (SPCD8). Expression was scaled per gene to highlight relative differences between populations. Corresponding log2 fold changes and adjusted p-values for highlighted genes are provided in Tables S6-S8. WT CD8^+^ T cells = WT splenic CD8^+^ T cells; ARE^−/−^ CD8^+^ T cells = ARE^−/−^ splenic CD8^+^ T cells. Data in bar plots represent mean ± SEM. Statistical comparisons were performed using Mann-Whitney tests unless otherwise indicated. *p < 0.05, **p < 0.01, ****p < 0.0001; ns, not significant.

Phenotypic and transcriptomic differences alone do not establish functional relevance. We therefore tested whether peripheral DNTs directly restrain CD8^+^ T-cell proliferation following stimulation. To do this, we performed coculture suppression assays using FACS-sorted splenic DNTs and CD8^+^ T cells. CFSE-labeled CD8^+^ T cells were stimulated with anti-CD3/CD28 and cultured alone or with DNTs at defined CD8^+^ T-cell: DNT ratios for 72 h, and proliferation was quantified by CFSE dilution and percent divided cells (Fig. 2A).

DNTs significantly suppressed proliferation of WT CD8^+^ T cells at both 2:1 and 1:1 CD8^+^ T cell: DNT ratios compared with stimulated CD8^+^ T cells cultured alone (Fig. 2B-C). To determine whether this suppression required direct cell contact, we repeated the assay using a transwell system that physically separated DNTs from CD8^+^ T cells while allowing exchange of soluble factors. Under these conditions, DNTs did not suppress CD8^+^ T-cell proliferation at either ratio tested (Fig. S3), consistent with a primarily contact-dependent mechanism under these conditions. We next asked whether DNTs also suppress CD8^+^ T cells isolated from an inflammatory setting. Using CD8^+^ T cells isolated from ARE^−/−^ mice (a model of chronic IFN-γ-driven inflammation)^10,11^, WT DNTs similarly suppressed CD8^+^ T-cell proliferation at both ratios tested (Fig. 2D-E), indicating that DNT-mediated inhibition extends to CD8^+^ T cells under inflammatory conditions. Because CD8^+^ T cells were stimulated polyclonally with anti-CD3/CD28, this assay measures generalized suppression of activated CD8⁺ T-cell proliferation rather than antigen-specific suppression. Representative post-sort purity and gating strategy for functional assays is shown in Fig. S4.

To examine effector output, we profiled cytokines in culture supernatants following anti-CD3/CD28 stimulation. Under these conditions, WT splenic DNTs (plated at 2.5 × 10^4^ cells per well due to lower cell yield) secreted IL-2 and IL-5 and low levels of IFN-γ, TNF-α, IL-6, IL-1β, IL-4, KC/GRO, and IL-10 (Fig. 2F). In contrast, WT splenic CD8^+^ T cells (plated at 5 × 10^4^ cells per well) stimulated under similar conditions produced high concentrations of IFN-γ, TNF-α, and IL-2, together with detectable KC/GRO (Fig. 2G). Though CD8^+^ T cells were plated at twice the number of DNTs, the difference in cytokine output exceeded this twofold difference in input cells. Thus, relative to CD8^+^ T cells, DNTs produced low levels of most measured cytokines, indicative of a constrained effector output rather than broad inflammatory secretion (Fig. 2F-G). Complementing these functional data, ex vivo RNA-seq analysis showed that splenic DN-enriched cells exhibit a checkpoint-rich regulatory/exhaustion-associated gene expression bias relative to splenic CD8^+^ T cells, including increased *Pdcd1*, *Lag3*, and *Tox*, alongside reduced *Prf1* and low inflammatory effector output in vitro (Fig. 2H; Table S7).

Together, these findings indicate that peripheral DNTs can directly restrain CD8^+^ T-cell proliferation through a primarily contact-dependent mechanism and exhibit reduced inflammatory effector output and a regulatory-biased/checkpoint and exhaustion transcriptional program.

### DNTs are transcriptionally distinct from CD8^+^ T cells, and ovarian DNT abundance tracks with the CD8 compartment in vivo

Having established that peripheral DNTs are enriched in the ovary and uterus and are phenotypically distinct from thymic DNTs, we next asked how DNTs relate transcriptionally to conventional CD8^+^ T cells and whether ovarian DNT abundance is linked to the CD8^+^ T-cell compartment in vivo. Prior work has shown that some TCRαβ DNT subsets can emerge in peripheral settings and retain partial overlap with CD8-associated programs^24–26,37,38^. We therefore first compared splenic and thymic DNTs and CD8^+^ T cells using RNA-seq to define broad transcriptional relationships among these populations before testing whether perturbation of the CD8 compartment alters ovarian DNT abundance.

**Fig. 3:**
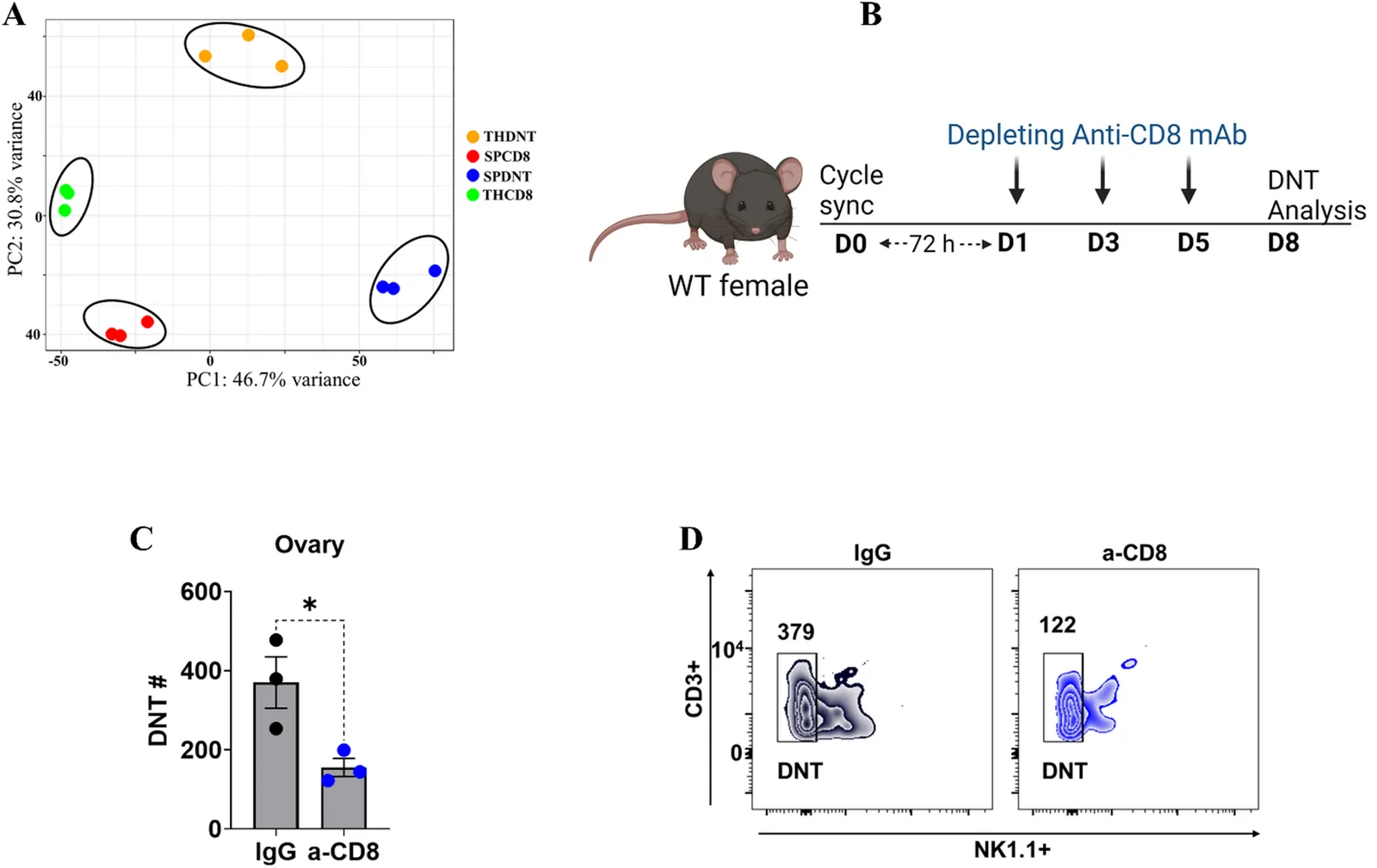
DNTs are transcriptionally distinct from CD8⁺ T cells, and ovarian DNT abundance is reduced by CD8-targeting depletion. (**A**) Principal component analysis (PCA) of RNA-seq data from splenic and thymic DNTs (SPDNT, THDNT) and CD8⁺ T cells (SPCD8, THCD8). Each point represents an individual sample (*n* = 3 biological replicates per group); PC1 separates DNTs from CD8⁺ T cells, whereas PC2 distinguishes splenic from thymic populations. (**B**) Experimental schematic for the in vivo CD8^+^ T-cell depletion. Estrous-synchronized WT females were treated with depleting anti-CD8 mAb or isotype IgG control on days 1, 3, and 5; tissues were harvested for DNT quantification on day 8. (**C**) Numbers of ovarian DNTs in IgG- versus anti-CD8-treated mice (*n* = 3 mice per group); Data are representative of two independent experiments. (**D**) Representative flow cytometry plots showing the ovarian DNT gate in IgG and anti-CD8 (a-CD8) groups, gated on live CD3^+^ lymphocytes and excluding NK1.1^+^ cells. Numbers shown in the representative flow plots indicate gated events and are shown for illustration only; ovarian DNT counts are quantified in the adjacent panel (**C**). Bars show mean ± SEM. Statistical comparisons between IgG and anti-CD8 groups were performed using unpaired two-tailed t tests; *p < 0.05.

Principal component analysis (PCA) separated the four groups into distinct clusters, with PC1 (46.7% variance) distinguishing DNTs from CD8^+^ T cells and PC2 (30.8% variance) further separating thymic from splenic samples (Fig. 3A). A focused heatmap of residency, and checkpoint-associated genes highlighted coordinated differences across these populations, including reduced *Cd8a*/*Cd8b1* and enrichment of checkpoint and survival-associated transcripts (*Pdcd1*, *Ctla4*, *Lag3*, *Tigit*, *Il2ra*)^39,40^ and (*Foxo1*, *Bcl2*, *Tox*)^41–43^ within DNTs relative to CD8^+^ T cells (Fig. S5; Table S8). Together, these analyses indicate that DNTs are transcriptionally distinct from conventional CD8^+^ T cells while retaining select T-cell signatures that vary with tissue context.

We next examined whether ovarian DNT abundance is linked to the CD8^+^ T-cell compartment in vivo. Estrous-synchronized WT females were treated with depleting anti-CD8 mAb or isotype IgG control on days 1, 3, and 5, and tissues were harvested for DNT quantification on day 8 (Fig. 3B). Anti-CD8 treatment reduced ovarian DNT absolute numbers relative to isotype controls (Fig. 3C-D). In contrast, uterine DNT numbers showed no clear change, and DNT numbers increased in lymph nodes and spleen following CD8^+^ T cell depletion (Fig. S6A-C), indicating that the CD8^+^ T-cell - DNT relationship is tissue dependent.

Because a small fraction of DNTs may retain very low CD8 expression, we cannot fully exclude some direct impact of anti-CD8 treatment on DNT recovery. However, the depletion-associated reduction was most evident in the ovary and was not mirrored in the uterus, LNs, or spleen (Fig. S6), making it less likely a uniform antibody effect on DNT quantification across tissues. Collectively, these data support a model in which ovarian DNT abundance is tightly associated with the CD8^+^ T-cell compartment, consistent with tissue-specific maintenance dynamics in reproductive versus lymphoid sites.

### Ovarian and uterine DNTs show limited exchange in parabiosis despite low expression of classical TRM markers

Having observed that ovarian DNT abundance is sensitive to CD8^+^ T-cell depletion, we next asked whether ovarian and uterine DNTs behave as locally maintained tissue pools or are replenished from the circulation. To address this, we assessed residency- and trafficking-associated markers and directly tested tissue exchange using parabiosis.

**Fig. 4:**
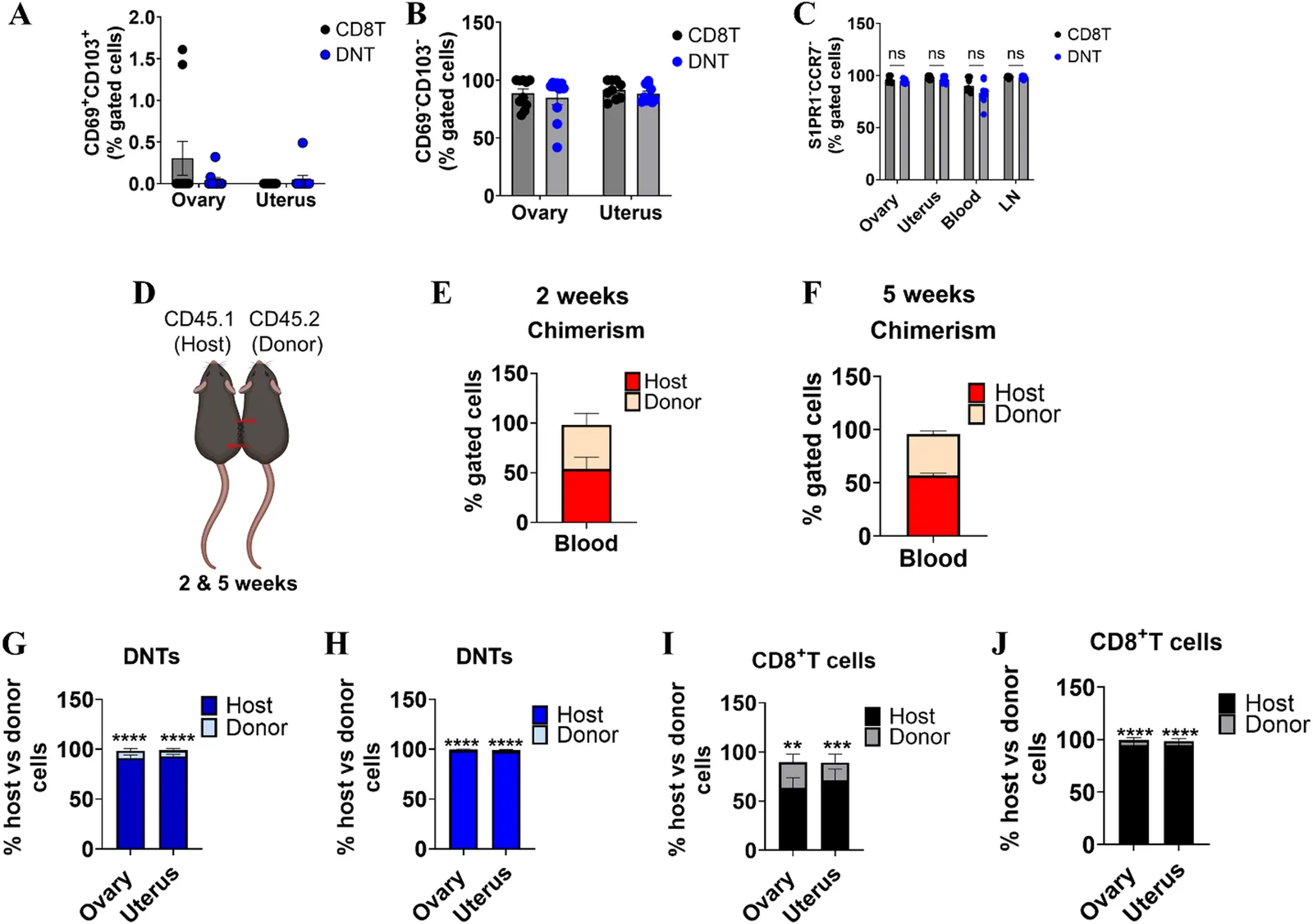
Ovarian and uterine DNTs lack classical tissue-resident markers and show limited exchange in parabiosis. (**A**, **B**) Frequencies of CD69⁺CD103⁺ (**A**), and CD69^−^CD103^−^ (**B**) cells among CD8^+^ T cells (CD8T, black) and DNTs (blue) in the ovary and uterus of WT females (*n* = 9 mice per group, Mann-Whitney test). (**C**) Frequencies of S1PR1^+^CCR7^+^ cells among CD8^+^ T cells and DNTs in ovary, uterus, blood, and lymph nodes (LN) (*n* = 6 mice per group, Mann-Whitney test). (**D**) Schematic of CD45.1 (host) x CD45.2 (donor) parabiotic female pairs, (**E**, **F**) peripheral blood chimerism at 2 weeks (**E**) and 5 weeks (**F**) after surgery. Bars show mean ± SEM percentage of host-derived versus partner-derived cells within the blood CD3^+^TCRβ^+^ T-cell gate. (**G**, **H**) Host-derived and partner-derived chimerism within ovarian and uterine DNT gates at 2 weeks (**G**) and 5 weeks (**H**) post-parabiosis. (**I**, **J**) Host-derived and partner-derived chimerism within ovarian and uterine CD8^+^ T-cell gates at 2 weeks (**I**) and 5 weeks (**J**) post-parabiosis. For (**G**, **J**), stacked bars represent the percentage of host-derived versus partner-derived cells within each tissue gate. Parabiosis data are from two independent cohorts (*n* = 5 pairs per time point). Bars show mean ± SEM. Statistical significance was assessed using Mann-Whitney tests; **p < 0.01, ***p < 0.001, ****p < 0.0001; ns = not significant.

Ovarian and uterine DNTs were largely negative for CD69 and CD103 (Fig. 4A-B and Fig. S7A). Though CD69 and CD103 are commonly used markers of tissue-resident memory (TRM) T cells^44,45^, their expression varies across non-lymphoid tissues and is not uniformly required for tissue retention, including at mucosal and reproductive sites^10,46–48^. Thus, the observed predominant CD69^−^CD103^−^ phenotype of ovarian and uterine DNTs is compatible with non-classical tissue adaptation and does not exclude local maintenance.

We next examined S1PR1 and CCR7, receptors that support tissue egress and lymphoid homing^49^, respectively. Across ovary, uterus, blood, and LNs, the dominant phenotype for both DNTs and CD8^+^ T cells was S1PR1^−^CCR7^−^, with few single-positive or double-positive subsets detectable (Fig. 4C and Fig. S7B-C). In agreement with this finding, S1PR1^+^ CCR7^+^ cells were relatively low within reproductive tissues compared with blood and lymphoid compartments (Fig. 4C), suggesting low frequency of a classical recirculating or lymphoid-homing phenotype in these tissue compartments.

To directly test tissue exchange, we paired congenically marked CD45.1 and CD45.2 females by parabiosis and assessed partner-derived contributions at two time points. Because blood chimerism is typically established by approximately two weeks^50,51^, the 2-week time point provides an early readout of tissue exchange following systemic equilibration. We also examined a 5-week time point, to allow recovery from the transient estrous disruption associated with parabiosis and to assess whether exchange patterns change over a longer interval^50^. Blood showed clear host and donor contributions at both 2 and 5 weeks (Fig. 4D-F), confirming successful parabiosis and systemic equilibration. In blood and spleen, partner-derived DNTs and CD8⁺ T cells were readily detectable (Fig. S7D-E), consistent with exchange through the circulation and lymphoid compartments.

Despite robust peripheral blood chimerism, ovarian and uterine DNTs remained host-derived at both 2 and 5 weeks, with minimal donor chimerism (Fig. 4G-H and Fig. S7F-G). Conversely, CD8^+^ T cells showed higher donor chimerism than DNTs at 2 weeks; however, by 5 weeks they were also strongly host-skewed in both ovary and uterus (Fig. 4I-J). These findings show limited exchange and slow replacement of local DNT and CD8^+^ T-cell pools from the circulation.

Overall, these findings indicate that ovarian and uterine DNTs exchange poorly between parabionts and are maintained predominantly as locally retained tissue pools, despite low expression of canonical CD69^+^CD103^+^ TRM markers.

### Chronic inflammation and infertility are associated with reduced DNT abundance in the ovary and uterus and improved pregnancy after DNT transfer

**Fig. 5:**
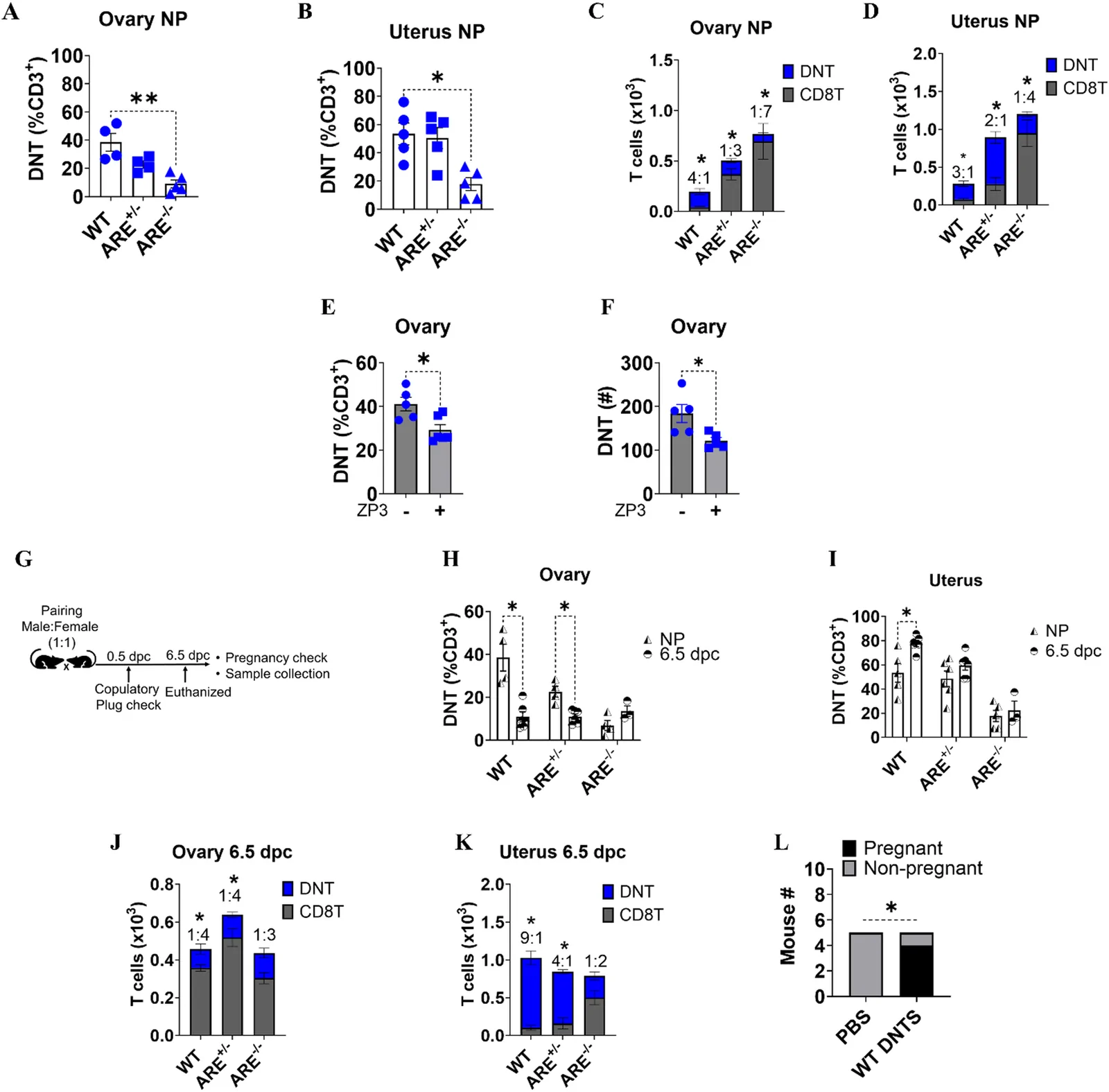
DNTs are reduced while CD8^+^ T cells expand in inflammatory reproductive dysfunction, and adoptive transfer of WT DNTs improves pregnancy outcome in ARE^−/−^ females. (**A**, **B**) Frequencies of DNTs among total CD3^+^ T cells in ovary (**A**) and uterus (**B**) from nonpregnant (NP) WT, ARE^+/−^, and ARE^−/−^ females at estrus (*n* = 4–5 mice per group). (**C**, **D**) Absolute numbers of DNTs (blue) and CD8^+^ T cells (gray) in ovary (**C**) and uterus (**D**); stacked bars indicate total CD3^+^ T cells, and DNT: CD8^+^ T cell ratios are shown above each bar (*n* = 4–5 mice per group). (**E**, **F**) Frequency of ovarian DNTs in WT females left untreated (ZP3^−^) or immunized with zona pellucida 3 peptide in complete Freund’s adjuvant (ZP3^+^) (**E**), and absolute ovarian DNT numbers in the same groups (**F**) (*n* = 5–6 mice per group). (**G**) Schematic of timed mating and tissue collection for early pregnancy analyses. WT, ARE^+/−^, and ARE^−/−^ females were paired with fertile WT males, checked for mating plugs at 0.5 days post coitum (dpc), and euthanized at 6.5 dpc for pregnancy assessment and tissue harvest. (**H**, **I**) Frequencies of DNTs in ovary (**H**) and uterus (**I**) from nonpregnant WT, ARE^+/−^, and ARE^−/−^ females at estrus (NP; triangles) and at 6.5 dpc (circles) (*n* = 3–6 mice per group). (**J**, **K**) Absolute numbers of DNTs (blue) and CD8^+^ T cells (gray) in ovary (**J**) and uterus (**K**) at 6.5 dpc; stacked bars indicate total CD3^+^ T cells, and DNT: CD8^+^ T cell ratios are shown above each bar (*n* = 3–6 mice per group). (**L**) Term pregnancy outcomes in plug-positive ARE^−/−^ females that received PBS or ex vivo FACS-sorted WT splenic DNTs 4 weeks prior to pairing (*n* = 5 mice per group). Bars indicate the number of pregnant (black) and nonpregnant (gray) females. This panel is shown as a stacked count/proportion plot summarizing pregnancy outcome per group and therefore does not include error bars. Data are presented as mean ± SEM except otherwise stated. Statistical comparisons were performed using Kruskal-Wallis tests where applicable; pregnancy frequencies were compared using Fisher’s exact test. *p < 0.05, **p < 0.01.

Given that peripheral DNTs can restrain CD8^+^ T-cell proliferation in vitro and exhibit a regulatory-biased program, we next asked whether the abundance of DNTs is altered in vivo in inflammatory settings linked to reproductive dysfunction. We first quantified DNTs in nonpregnant WT, ARE^+/−^, and ARE^−/−^ females at estrus. In ARE^−/−^ mice, which develop chronic IFN-γ-driven inflammation and reproductive dysfunction^10,11^, DNT frequencies were reduced in both the ovary and uterus compared with WT and ARE^+/−^ controls (Fig. 5A-B). This reduction was accompanied by increased CD8^+^ T-cell abundance, resulting in lower DNT: CD8⁺ T-cell ratios in both tissues (Fig. 5C-D). However, DNT frequencies in lymphoid tissues were comparatively preserved across genotypes with differences seen primarily in blood (Fig. S8A-C), indicating that the DNT deficiency is most pronounced at reproductive sites under inflammation.

To test whether ZP3-immunization similarly affects ovarian DNT abundance, we examined WT females immunized with zona pellucida 3 (ZP3) peptide in complete Freund’s adjuvant (CFA), a model associated with ovarian inflammatory pathology^8,52^. ZP3-CFA immunization reduced ovarian DNT frequencies relative to unimmunized controls and was also associated with reduced absolute ovarian DNT number (Fig. 5E-F and Fig. S8D), supporting reduced ovarian DNT abundance in this setting. We next assessed how these shifts present during early pregnancy by analyzing tissues at 6.5 days post coitum (dpc) (Fig. 5G). Across genotypes, DNT frequencies were lower at 6.5 dpc than in the nonpregnant state, with ARE^−/−^ females showing the lowest DNT abundance in both ovary and uterus (Fig. 5H-I). Absolute counts at 6.5 dpc showed persistent CD8^+^ T-cell predominance and a reduced DNT: CD8^+^ T-cell ratio in the ovary and uterus of ARE^+/−^ and ARE^−/−^ mice relative to WT (Fig. 5J-K). Concordant with a more activated CD8^+^ T-cell compartment in the inflammatory genotypes, ARE^+/−^ and ARE^−/−^ females showed increased effector/effector-memory-like CD8^+^ T cells in the ovary and uterus at 6.5 dpc compared with WT (Fig. S8E-G).

To test whether augmenting the DNT compartment can improve fertility in the context of chronic IFN-γ-driven inflammation, we assessed term pregnancy outcomes following adoptive transfer of WT DNTs into ARE^−/−^ females. This experiment was not designed to determine whether transferred DNTs preferentially localize to reproductive tissues or directly alter local CD8⁺ T-cell abundance or activation after transfer. ARE^−/−^ females received ex vivo FACS-sorted splenic WT DNTs four weeks prior to pairing, and pregnancy outcomes were assessed among plug-positive females. WT DNT transfer increased the fraction of females that achieved pregnancy compared with PBS-treated controls (Fig. 5L). Together, these data show that inflammatory reproductive dysfunction is associated with a marked reduction of DNTs in the ovary and uterus alongside CD8^+^ T-cell expansion, and they provide functional evidence that augmenting peripheral DNTs can improve pregnancy outcome in ARE^−/−^ females.

## Discussion

This study defines a conventional NK1.1⁻ TCRβ^+^ double-negative T cells (DNTs) compartment across lymphoid and reproductive tissues and examines how it changes in inflammatory settings associated with impaired fertility. Across two complementary models, we observe a consistent pattern within reproductive tissues: the DNT compartment decreases while CD8^+^ T cells increase and display a more activated phenotype, shifting the local DNT: CD8^+^ T cell balance toward effector predominance. In parallel, ex vivo FACS-sorted splenic DNTs suppress CD8^+^ T cell proliferation largely through contact-dependent mechanisms, supporting the concept that peripheral DNTs can provide regulatory restraint that may be relevant within tissue microenvironments where close-range immune interactions occur. Together, these findings position the DNT: CD8^+^ T cell relationship as a measurable outcome of inflammatory reproductive dysfunction.

The DNT compartment described here shares features with DNT populations implicated in immune regulation in autoimmunity, transplantation, and cancer, while extending these observations to reproductive tissues from nonpregnant mice. Prior studies have shown that TCRαβ^+^ DNTs can suppress effector T cell responses and influence antigen-presenting cell function in a range of inflammatory contexts^20,53–56^. In humans, decidual DNTs are enriched at the maternal-fetal interface and have been associated with fetal tolerance and protection from recurrent pregnancy loss^23^. Building on these observations, our data provide phenotypic characterization of TCRβ^+^NK1.1^−^ DNTs across female tissues, alongside transcriptomic characterization of a spleen- and thymus-derived NK1.1^−^DNT-enriched population in the ovary and uterus, and demonstrate that DNT loss accompanies inflammatory conditions that impair fertility.

Functional assays support a regulatory-biased/non-canonical suppressive state program in peripheral DNTs. Compared with CD8^+^ T cells, DNTs show reduced cytotoxic effector programs and increased checkpoint-associated features, consistent with a differentiated state and reduced cytolytic output. In vitro, FACS-sorted splenic DNTs produced relatively low inflammatory cytokine output after CD3/CD28 stimulation and suppressed CD8^+^ T-cell proliferation. Our findings also showed that suppression was diminished when DNTs were separated by a transwell barrier, indicating that regulation is primarily contact-dependent and likely requires cell-to-cell proximity. This contact dependence suggests that DNT-mediated restraint is spatially constrained. These functional assays were performed using splenic DNTs for practical yield reasons. Accordingly, they define a peripheral suppressive capacity of TCRβ^+^NK1.1^−^ DNTs under activating conditions, but do not by themselves establish that ovarian or uterine DNTs exert identical suppressive activity in vivo. Though reproductive-tissue DNTs shared selected phenotypic features with peripheral DNTs, direct transcriptomic profiling was not performed on ovarian or uterine DNTs in this study. Accordingly, splenic DNTs should be viewed as a practical peripheral reference population not as fully equivalent to reproductive-tissue DNTs, which may acquire additional context-dependent programs in the ovary and uterus.

An important interpretive point is that the checkpoint-associated transcriptional features observed in the NK1.1^−^DNT-enriched RNA-seq dataset, including elevated *Pdcd1*, *Lag3*, and *Tox*, are not uniquely regulatory and may also overlap with programs associated with chronic stimulation or T-cell exhaustion^57,58^. Our data therefore support a checkpoint-rich, low-effector state that is compatible with suppressive function in vitro. This interpretation is further supported by the combination of reduced *Prf1*, lack of clear *Gzmb* upregulation in splenic DNTs, low inflammatory cytokine output after CD3/CD28 stimulation, and absence of a canonical Foxp3-centered Treg transcriptional program. At the same time, the possibility that some of these cells arise through chronic stimulation or peripheral adaptation from conventional CD8^+^ T-cell states remains open and deserves direct lineage-focused investigation.

A central observation is the reciprocal relationship between DNTs and CD8^+^ T cells in inflamed reproductive tissues. In both the ARE^−/−^ and ZP3-CFA models, DNT frequencies decrease in the ovary and uterus while CD8^+^ T cells expand, lowering the DNT: CD8^+^ T-cell ratio and increasing effector/effector-memory-like CD8^+^ T-cell phenotypes. These changes occur in settings where CD8^+^ T cells and IFN-γ have been implicated in autoimmune oophoritis and chronic IFN-γ-driven reproductive dysfunction^8–11,52^, and they are not mirrored to the same extent in lymphoid compartments, where DNT frequencies are comparatively preserved. This tissue selectivity suggests that DNTs in reproductive sites are disproportionately impacted by inflammatory cues, whether through reduced persistence, altered recruitment, or impaired tissue retention, in parallel with conditions that favor effector CD8^+^ T-cell accumulation. Within this framework, DNTs may complement established regulatory pathways to dampen local effector CD8^+^ T-cell activity in reproductive tissues. Loss of this DNT compartment, could therefore contribute to an effector-skewed state that reduces tissue tolerance in inflammation-associated infertility.

Our parabiosis data points to limited exchange of ovarian and uterine DNTs. Despite low expression of classical CD69 and CD103 TRM markers, ovarian and uterine DNTs showed minimal partner-derived chimerism over 2 to 5 weeks, supporting the presence of a locally retained or slowly exchanging tissue pool. Importantly, low partner contribution can reflect true tissue retention, slow baseline turnover, restricted vascular access, or an equilibration window that exceeds the parabiosis interval. Therefore, we interpret these data as evidence of limited exchange rather than definitive proof of a classical tissue-resident memory (TRM) identity. This distinction is particularly relevant in reproductive tissues, where resident-like immune programs may diverge from CD69/CD103-defined TRM phenotypes described in other organs^10,44–46^. Further work using complementary strategies, including intravascular labeling, longer-term parabiosis, and lineage approaches, will be needed to clarify the mechanisms that regulate DNT persistence, trafficking, and exchange in reproductive sites.

Our findings also supported a regulatory-biased/ exhaustion-associated program in peripheral DNTs. DNTs retain core TCR-associated transcripts and exhibit a CD44^hi^ antigen-experienced phenotype, yet diverge from conventional CD8^+^ T cells through reduced cytotoxic effector programs and increased expression of inhibitory receptors and regulatory-associated genes. Consistent with this tissue-tuned state, phenotypic profiling across tissues highlighted differences in TCR-associated features and differentiation markers. Relative to tissue-matched CD8^+^ T cells, DNTs showed distinct patterns of CD5 and TCRβ expression across lymphoid and reproductive tissues, supporting the concept that local cues may tune activation thresholds and signaling capacity. In parallel, Runx3 and T-bet profiling further positioned reproductive-tissue DNTs within a differentiated immune landscape, and supports a transcriptomic difference between SPDNT and SPCD8 populations. Cumulatively, these features reflect a tissue-adapted state influenced by local cues. Though reports in other systems suggest that some TCRαβ DNT subsets can emerge through peripheral adaptation from conventional T-cell states under specific activation conditions^24–29^, our data do not directly establish developmental relationships between reproductive-tissue DNTs and CD8^+^ T cells. However, our results support a coordinated association between these compartments, highlighted by suppression of CD8^+^ T-cell proliferation in vitro, tissue-specific shifts in the DNT: CD8^+^ T-cell balance in vivo, and the selective reduction of ovarian DNTs following CD8^+^ T-cell depletion.

The adoptive transfer data in infertile ARE^−/−^ females provide functional support that augmenting the DNT compartment can improve reproductive outcome in a chronically inflamed setting. Transfer of ex vivo FACS-sorted WT splenic DNTs increased pregnancy frequency relative to controls. These findings do not define the direct cellular targets of DNT activity in vivo or establish DNTs as a standalone therapeutic approach, neither do they define whether transferred DNTs home to reproductive tissues, persist locally, or directly suppress local CD8⁺ T-cell responses in vivo. However, they support a model in which augmenting the peripheral DNT compartment can improve pregnancy outcome in a chronically inflamed setting^59,60^, though whether this involves changes in the local immune composition of reproductive tissues remains to be determined. Classical CD4^+^ Foxp3^+^ Tregs remain the best-established T-cell mediators of implantation and maternal-fetal tolerance, and their numerical or functional impairment is strongly linked to reproductive failure. In mice, depletion or disruption of Foxp3^+^ Tregs compromises implantation and promotes a hostile uterine microenvironment, while peri-implantation uterine Treg accumulation is shaped by seminal and local reproductive cues. In contrast, the DNT population described here likely represents a non-classical, Foxp3-independent suppressive compartment that may overlap functionally with Tregs in limiting inflammatory T-cell activity, but differs in phenotype and lineage-defining markers^61–64^.

This study has some limitations, and several considerations structure the scope of this work and inform next steps. First, these studies were performed in mouse models, and defining how closely these DNT-CD8^+^ T-cell relationships map onto human ovarian and uterine immune regulation will be an important future direction. Second, limited DNT yield from ovary and uterus constrained deeper transcriptomic and mechanistic assays in those tissues, and we therefore leveraged splenic DNTs as a practical model to define core peripheral DNT features. While splenic and reproductive-tissue DNTs share selected phenotypic features, reproductive-tissue DNTs may also acquire additional context-dependent programs that were not resolved here. An important limitation of the transcriptomic analysis is that the RNA-seq sort used a reduced DN-enrichment strategy without direct TCRβ or TCRγδ discrimination, and it did not specifically exclude MAIT cells. Accordingly, transcriptomic data are best interpreted as describing an NK1.1^−^ DNT cell-enriched compartment. Third, our data support coordinated behavior between DNTs and CD8^+^ T cells based on suppression assays, transcriptional features, tissue-specific shifts in the DNT: CD8^+^ T-cell ratio, and sensitivity of ovarian DNT abundance to CD8^+^ T-cell depletion; however, developmental relationships cannot be inferred without lineage-based strategies, and we cannot fully exclude minor effects of anti-CD8 treatment on recovery of rare CD8^lo^ DNT events. We also did not include CD4^+^Foxp3^+^ Tregs as a benchmark suppressor population in the in vitro coculture assays, and therefore the relative suppressive potency of DNTs versus canonical Tregs was not addressed in this study. Fourth, the pregnancy transfer experiments focused on the ARE^−/−^ model and were powered to detect differences in term pregnancy frequency; they were not designed to assess post-transfer DNT localization, persistence within reproductive tissues, or local effects on CD8^+^ T-cell composition or activation. Thus, expanding cohort sizes and incorporating complementary endpoints (e.g., implantation and resorption indices, litter size, fetal viability, and tissue pathology) will better define the magnitude of the effect and the immune changes associated with rescue. Furthermore, analyses were performed at estrus in nonpregnant mice and a single early pregnancy time point (6.5 dpc); extending sampling across the estrous cycle and additional gestational windows will provide a more complete view of temporal immune adaptation in reproductive tissues.

In summary, we define a reproductive tissue-enriched NK1.1^−^ TCRβ^+^ DNT compartment with a contact-dependent capacity to restrain CD8^+^ T-cell proliferation. We show that DNT abundance and the local DNT: CD8^+^ T-cell ratio are selectively disrupted in inflammatory models associated with reproductive dysfunction, and that augmenting the peripheral DNT compartment improves pregnancy frequency in a chronically inflamed IFN-γ-elevated setting. Together, these findings identify the DNT: CD8^+^ T-cell axis as a candidate immunoregulatory feature of reproductive tissues that is perturbed during inflammatory reproductive dysfunction.

## Methods

### Animals

Female C57BL/6J mice were used in this study, including wild-type (WT; JAX stock #000664) and mice heterozygous or homozygous for the interferon-γ AU-rich element replacement (ARE^+/−^ and ARE^−/−^), aged 3–5 months. The generation of ARE mice has been described previously^10,11^. Briefly, a 162-nucleotide AU-rich element sequence within the 3′ untranslated region (3′UTR) of the *Ifng* gene was excised using the Cre/lox system and replaced with a neutral sequence by electroporation into embryonic stem cells. The modified locus was used to generate chimeric mice, and independent ARE lines were backcrossed for more than ten generations onto the C57BL/6 background^10,11^. Genotyping was performed as detailed in the Supplementary Methods.

Mice were housed and bred at the National Cancer Institute-Frederick animal facility with a 12 h light-dark cycle and *ad libitum* access to food and water. Unless otherwise specified, females were weighed and their estrous stage determined by vaginal lavage on the day of experimentation (see estrous cycle staging section). Mice were euthanized under isoflurane anesthesia, and ovaries, uteri, spleen, and peripheral lymph nodes were collected for downstream flow cytometry, RNA sequencing, and cytokine analyses. Whole blood was obtained by cardiac puncture for plasma preparation. All animal procedures were conducted in accordance with the National Institutes of Health Guide for the Care and Use of Laboratory Animals and were reported in compliance with the ARRIVE guidelines protocols. The protocols were approved by the National Cancer Institute Animal Care and Use Committee (NCI ACUC, Frederick, MD; protocol# 21 − 018 and Loyola University Chicago, protocol # 213977).

### Estrous cycle staging

Estrous cycle stage was assessed by vaginal lavage (VL) as described previously^65,66^. Additional information regarding the phases of the estrous cycle can be found in the supplementary materials. A volume of 50–100 µl of VL was applied to clean microscope slides (Corning, USA) and left to air-dry at room temperature. Once dry, the slides were fixed using cold 100% methanol and stained with a 0.01% solution of methylene blue (Sigma Aldrich, USA)^66^ before being examined under a microscope to identify the cycle stages. To synchronize the estrous cycles, the females were housed in beddings previously infused with male urine for 48 h prior to the experiments^67^, and VL samples were collected at the conclusion of the experiments to assess cycle stages.

### Mating and pregnancy assessment

Fertility and early pregnancy outcomes were assessed in WT and ARE^−/−^ females by timed mating with fertile WT C57BL/6J males. Female mice were mated with at a pairing ratio of 1:1 (female: male). The females were monitored for the presence of mating plugs beginning the morning following pairing. The presence of a mating plug was recorded as 0.5 days post coitum (dpc). Plug-positive females were either euthanized at 6.5 dpc for analysis of early pregnancy or followed longitudinally for assessment of pregnancy frequency. For 6.5 dpc cohorts, uteri were dissected and examined for embryo implantation sites. For longer-term fertility assays, plugged females were monitored for up to 22 days for evidence of pregnancy and parturition. Pregnancy frequency was defined as the proportion of plugged females that took pregnancies to term or delivered at least one litter within the observation period.

### Tissue processing and single-cell preparation

On the day of experimentation, mice were euthanized under isoflurane anesthesia and ovaries, uteri, spleen, and peripheral lymph nodes (LNs) (including LNs draining the ovary and uterus) were carefully dissected, and weighed. Tissues were transferred into cold Roswell Park Memorial Institute (RPMI) 1640 medium (Corning) supplemented with 10–20% fetal bovine serum (FBS), 100 U/ml penicillin, 10 µg/ml streptomycin, and 2 mM L-glutamine.

For reproductive tissues, fat and connective tissues were removed from the ovaries and uteri before transfer into a digestion mixture containing 0.01 mg/ml collagenase type IV (Sigma-Aldrich, USA). In some preparations, 4 U of DNase I (Sigma-Aldrich, USA) were added to reduce clumping, and the total volume was adjusted to 5 ml with supplemented RPMI. Ovaries and uterine horns were then sliced into small fragments and digested in a gentleMACS Octo Dissociator with heaters (Miltenyi Biotec, USA) using a custom program (37 °C for 20 min at 400 rpm). Immediately after digestion, the cell suspension was placed on ice and additional RPMI was added to halt the enzymatic reaction. Digested suspensions were passed through a 40 μm cell strainer, washed with RPMI/FBS, and centrifuged at 1,200 rpm for 5 min at 4 °C. Where necessary, red blood cells were lysed using ACK lysing buffer (Quality Biological) for 5 min at room temperature, followed by quenching with excess phosphate-buffered saline (PBS) and washing.

Spleens and LNs were processed by gentle mechanical dissociation in a Filtra blender bag (Labplas) as previously described^66^. Briefly, each spleen or LNs per mouse was placed in one pouch of the Filtra bag containing approximately 3 ml RPMI, gently mashed, and rinsed with an additional 10 ml PBS. The cell suspension was recovered from the opposite pouch, transferred to a 50 ml tube, centrifuged at 1,200 rpm for 5 min at 4 °C, and resuspended in 5 ml ACK lysing buffer (Quality Biological) for 5 min at room temperature. Lysis was stopped with 20 ml PBS, followed by centrifugation and resuspension in FACS buffer; the suspension was then filtered through a 40 μm cell strainer.

The pellets obtained were resuspended in cold FACS-buffer and counted for further antibody staining as necessary^66^ (Table S1) or for further downstream assays. Leukocyte counts were performed using a Sysmex KX-21 N Hematology Analyzer, while total cell counts were obtained utilizing a Cellometer Auto T4 (Nexcelom Bioscience, USA).

### Flow cytometry and cell sorting

Cell pellets from the ovary, uterus, blood, LNs, and spleen (typically 2–5 × 10^6^ cells) were prepared in 5 ml tubes for flow cytometry. Dead cells were excluded using Zombie Aqua fixable viability dye (BioLegend, San Diego, CA) applied at a 1:50 dilution; cells were incubated for 30 min at room temperature in the dark, then washed with FACS buffer (PBS containing 2% FBS and 2 mM EDTA). Pellets were resuspended in 20 µl of a 1:10 dilution of Fc block (anti-CD16/CD32, clone 2.4G2, BD Biosciences, San Jose, CA) and incubated for 10 min at 4 °C in the dark. Fluorochrome-conjugated antibodies (see Table S1 for full panel) were added in the presence of Fc block and incubated for 30 min at 4 °C in the dark for surface staining. After 30 min, cells were washed with FACS buffer and centrifuged at 1,500 rpm for 5 min at 4 °C, and the final pellets were resuspended in FACS buffer for acquisition.

Controls included unstained samples, single-stained compensation beads, and single-stained cell samples for setting PMT voltages and compensation. Fluorescence-minus-one (FMO) controls were used to aid in gate placement. Data were acquired on BD LSRFortessa, or BD FACSymphony A5, and BD FACSymphony S6 Cell sorter (BD Biosciences) using FACSDiva software and analyzed with FlowJo software (version 10.10.0, BD Biosciences, USA).

For analysis, debris and unlysed erythrocytes were excluded based on FSC-A versus SSC-A profiles, and doublets were removed by FSC-A versus FSC-H gating. Live CD45^+^ events were then gated before further subdivision. CD8^+^ T cells were defined as live CD45^+^CD3^+^TCRβ^+^CD8α^+^CD4^−^ cells. Double-negative T cells (DNTs) were defined as live CD45^+^CD3^+^TCRβ^+^CD8α^−^CD4^−^NK1.1^−^ cells. Effector-like CD8^+^ T cells were defined as CD44^hi^CD62L^lo^ and naive CD8^+^ T cells as CD44^lo^CD62L^hi^ within the CD8^+^ gate. For sorting used in functional assays and adoptive transfer experiments, DNTs and CD8^+^ T cells were purified using the same TCRβ-based gating strategy; sorted cells were collected into tubes containing RPMI with 20% FBS, and post-sort purity was routinely > 90% (Fig. S4).

### In vitro proliferation and suppression assays

Cells were cultured in RPMI 1640 medium (Thermo Fisher Scientific) supplemented with non-essential amino acids, 1 mM sodium pyruvate, 10 mM HEPES, 100 U/ml penicillin, 100 µg/ml streptomycin (Thermo Fisher Scientific), 50 µM 2-mercaptoethanol, 2 mM L-glutamine (Sigma-Aldrich), and 10% FBS (VWR). Cultures were maintained at 37 °C in a humidified incubator with 5% CO₂.

For suppression and viability assays, sorted DNTs and splenic CD8^+^ T cells were cultured either alone or in direct-contact coculture at defined CD8^+^ T cell: DNT ratios. Briefly, 5 × 10^4^ splenic CD8^+^ T cells were plated alone (1:0) or together with 2.5 × 10^4^ (2:1) or 5 × 10^4^ (1:1) splenic DNTs in flat-bottom 96-well plates in the presence of 2 µl anti-CD3/CD28 Dynabeads (Thermo Fisher Scientific) per well. CD8^+^ T cells, and where indicated DNTs, were labeled with CFSE or CellTrace Yellow/Violet (Thermo Fisher Scientific) prior to plating according to the manufacturer’s instructions to monitor proliferation. Cultures were set up in technical duplicates and incubated for 72 h. At the end of culture, cells were harvested for flow cytometric analysis of proliferation and viability, and supernatants were collected and stored at -80 °C for cytokine measurements. Because activation was induced with anti-CD3/CD28 Dynabeads alone, these coculture assays assess generalized suppressive effects on activated CD8^+^ T-cell proliferation and not antigen-specific suppression.

### Cytokine measurements

Cytokines in cell culture supernatants were quantified using electrochemiluminescence-based multiplex assays (Meso Scale Discovery, MSD). Mouse multiplex kits measuring 10 or 19 analytes (catalog numbers C4048-1; Meso Scale Discovery) were used according to the manufacturer’s instructions. Supernatants were thawed on ice, diluted as required, and assayed in duplicate on pre-coated multi-spot plates. Plates were read on a QuickPlex SQ 120 instrument (MSD), and analyte concentrations were calculated from standard curves using Discovery Workbench 4.0 software. Technical replicates were averaged and multiplied by the appropriate dilution factor. Final cytokine concentrations were reported in pg/ml and are presented as mean ± SEM.

### ZP3-induced autoimmune ovarian inflammation

Antigen-specific ovarian inflammation was induced by immunization with murine zona pellucida 3 (ZP3) peptide emulsified in Freund’s adjuvant. Briefly, a 1 mM stock of murine ZP3 peptide (ZP3^330–342^ NSSSSQFQIHGPR/GenScript) was emulsified 1:1 (v/v) in CFA (Sigma Aldrich, USA) by vigorous homogenization to generate a stable water-in-oil emulsion. For immunization, the ZP3-CFA emulsion was diluted with PBS to a final peptide concentration of 50 nM, and 50 µl of this emulsion was injected subcutaneously into female C57BL/6J mice aged 3–4 months that had been anesthetized with isoflurane according to approved ACUC protocols. Age-matched C57BL/6J females that did not receive any injection served as untreated controls. Fourteen days after immunization (day 15), mice were euthanized and blood, ovaries, uteri, spleen, and LNs were collected as described above. Reproductive tissues were processed into single-cell suspensions and analyzed by flow cytometry to quantify DNTs and CD8^+^ T-cells.

### Parabiosis

Parabiosis was performed to assess the recirculation and tissue residency of DNTs and CD8^+^ T cells as previously described^68^. Briefly, age-matched female C57BL/6J mice congenic for CD45.1 and CD45.2 were paired and co-housed for 3 days before surgery. On the day of surgery, the corresponding right or left lateral aspect of each parabiont was shaved, and mice received a subcutaneous injection of extended-release buprenorphine (0.5-1 mg/kg) for analgesia. Under isoflurane anesthesia, a longitudinal skin incision was made from the olecranon to the knee of each parabiont, and the subcutaneous fascia was bluntly dissected to free the skin. The olecranons and knees of each pair were sutured together using absorbable chromic gut suture (Covidien; 4 − 0 UG-203), and the dermis of the two parabionts was apposed and closed with wound clips to establish a shared circulatory system.

Post-operative care included administration of 5% dextrose in 0.9% sodium chloride, provision of moist food on the cage floor, and antibiotics (Enroquin, Dechra) in the drinking water for 1 week. Parabiotic pairs were maintained for 2–5 weeks to allow equilibration of circulating leukocytes. At the indicated time points (2 and 5 weeks), mice were euthanized and blood, ovaries, uteri, spleen, and LNs were harvested. Single-cell suspensions were prepared as described in Sect.  2.4 and stained for CD45.1, CD45.2, and T cell markers (Sect.  2.5). Chimerism within DNTs and CD8^+^ T-cells, was quantified as the proportion of partner-derived (discordant CD45 allotype) versus host-derived cells in each tissue. Parabiosis data were from two independent cohorts with *n* = 5 parabiotic pairs per cohort. Successful parabiosis was confirmed by the presence of greater than 40% partner-derived cells in peripheral blood at each timepoint prior to tissue analysis. Pairs that failed to achieve this chimerism threshold or developed post-surgical complications requiring early termination were excluded from analysis. Vaginal lavage was performed at the time of tissue harvest to document estrous cycle stage in parabionts.

### Adoptive transfer of DNTs in ARE^−/−^ females

Adoptive transfer experiments were performed to assess whether augmenting the DNT compartment could influence pregnancy outcomes in IFN-γ-driven reproductive dysfunction. DNTs were sorted from spleens of female WT C57BL/6J mice using the gating strategy described in “Flow cytometry and cell sorting section”. Sorted cells were washed and resuspended in sterile PBS.

Female ARE^−/−^ mice aged 3–4 months were randomized to the WT DNT transfer or control group. Mice in the DNT group received 2 × 10^5^ DNTs in a total volume of 100 µl PBS by intravenous tail-vein injection, administered 4 weeks before pairing with WT C57BL/6J males for timed mating. Control ARE^−/−^ mice received an equal volume of PBS alone.

Four weeks after transfer, females were placed in urine-infused spent male bedding 48 h before pairing with fertile WT males, and the presence of a mating plug the next morning was recorded as 0.5 dpc. Plug-positive females were then monitored for 21–22 days for evidence of pregnancy and parturition. Pregnancy frequency was defined as the proportion of plugged females that carried a pregnancy to term within the observation period. Data presented were recorded from ARE^−/−^ females with confirmed IFN-γ elevation characteristic of the ARE^−/−^ mouse model.

### Intravascular CD45 labeling

Intravascular antibody labeling was used to distinguish circulating from tissue-compartment leukocytes. Anti-CD45.1 (clone A20, BioLegend) or anti-CD45.2 (clone 104, BioLegend) antibodies were prepared under sterile conditions and diluted in sterile 1x PBS at 4 °C at 1:100 dilution (3 µg). For intravascular labeling, 300 µl of diluted anti-CD45.1 or anti-CD45.2 antibody was injected intravenously via the tail vein into anesthetized mice. Three minutes after injection, mice were euthanized and blood, ovaries, uteri, spleen, and LNs were collected and processed into single-cell suspensions as described in section on tissue processing and single cell preparation. Labeled cells were detected by flow cytometry, and circulating versus tissue-compartment populations were distinguished based on the presence or absence of the injected CD45.1/CD45.2 signal within each gated T cell subset. For parabiosis experiments, each parabiont was intravenously labeled with the antibody specific for its own congenic genotype (anti-CD45.1 or anti-CD45.2), immediately prior to euthanasia^69^.

### RNA sequencing and bioinformatic analysis

For transcriptomic profiling, a DN T-cell-enriched population and CD8^+^ T cells were FACS-sorted from spleen and thymus. To maximize recovery from low abundance DN populations during pilot efforts to obtain transcriptomic material from reproductive tissues, a reduced antibody panel was used for this sort. Even after pooling ovary and uterus samples, reproductive-tissue DN yields remained insufficient for sequencing, and RNA-seq was therefore continued on spleen and thymus cells using this reduced DN-enrichment strategy.

Live CD3^+^CD8α^−^CD4^−^NK1.1^−^ cells were collected as the DN-enriched population, and live CD3^+^ CD4^−^CD8α^+^ cells were collected as CD8^+^ T cells. Because TCRβ or TCRγδ was not included in the RNA-seq sort panel, these transcriptomic data should be interpreted as deriving from an NK1.1^−^ DN T-cell-enriched population. For each population and tissue, *n* = 3 biological replicates were generated, each replicate pooled from 20 female WT C57BL/6J mice aged 3 months cycle synchronized by PMSG (i.p., 100 µL/mouse) 48 h prior to harvest. Each pooled replicate yielded approximately 1–2 × 10^6^ cells. Pooling was required to obtain sufficient numbers of DN-enriched cells for robust RNA yield and library preparation. Total RNA was isolated using the RNAqueous-Micro Kit (Thermo Fisher Scientific, AM1931) following the manufacturer’s protocol including DNase treatment. RNA quantity and integrity were assessed by fluorometric measurement and Agilent Bioanalyzer; only samples with adequate yield and integrity were taken forward for library preparation.

Libraries were prepared with the Illumina Stranded Total RNA Prep, Ligation with Ribo-Zero Plus kit and sequenced as total RNA-seq on an Illumina NextSeq 2000 P2 flow cell (paired-end, read length 51 bp for R1 and R2). Twelve libraries were pooled and sequenced together. Each sample yielded 67–97 million passing-filter reads with more than 94% of bases at Q30 or higher. After alignment, the average mapping rate was 89%, uniquely mapped reads were above 64%, unmapped reads ranged from 7.02 to 28.70%, ribosomal bases averaged 2.07%, coding bases 14–35%, UTR bases 22–45%, and mRNA bases 39–72%, with 23–69% non-duplicate fragments as assessed by Picard’s MarkDuplicates and RNA-seq metrics.

Reads were trimmed for adapters and low-quality bases using Cutadapt, then aligned to the mouse genome (mm10) using STAR. Gene-level quantification was performed with RSEM to generate raw and normalized counts. The resulting count matrix (DNTSPTHY RawCountFile_rsemgenes.txt) was imported into R (version 4.5.2) using RStudio (version 2025.9.1.0) for downstream analysis with DESeq2 (version 1.50.2). Lowly expressed genes were filtered, counts were normalized with the median-of-ratios method, and differential expression was assessed between DNT and CD8^+^ T-cell populations within each tissue. Log2 fold changes were shrinkage-estimated and p-values were adjusted by the Benjamini-Hochberg procedure; genes with padj < 0.05 were considered differentially expressed unless stated otherwise. Variance-stabilized or regularized log-transformed counts were used for principal component analysis. Functional enrichment analysis was performed in R using Gene Ontology (GO) Biological Process terms and KEGG pathway gene sets^70,71^, and results were visualized as ggplot2 dot plots showing gene ratio and adjusted p-values. KEGG analyses used KEGG pathway gene sets for enrichment testing only; no KEGG pathway map images were reproduced. Heatmaps were generated with the pheatmap package, with rows scaled to z-scores where indicated, and PCA plots were produced using ggplot2.

### Statistical analysis

Statistical analyses were performed using GraphPad Prism (version 10.6.1, GraphPad Software, San Diego, CA) and R (version 4.5.2). Data are presented as mean ± SEM unless otherwise indicated. For comparisons between two groups, non-parametric Mann-Whitney tests were used; otherwise, unpaired two-tailed Student’s t tests were applied. For experiments involving more than two groups, Kruskal-Wallis test was used; otherwise one-way or two-way analysis of variance (ANOVA) with Tukey’s post hoc multiple-comparison tests was used as appropriate. Pregnancy frequencies were compared using Fisher’s exact test. P values < 0.05 were considered statistically significant. Adjusted p values for RNA-seq differential expression were calculated using the Benjamini-Hochberg procedure. All statistical tests and the number of biological replicates (n) are reported in the figure legends or main text.

## Supplementary Information

Below is the link to the electronic supplementary material.

Supplementary Material 1

## Data Availability

RNA-seq data have been deposited in Gene Expression Omnibus (GEO) under accession GSE317190. Other data are available from the corresponding author upon reasonable request.
