# Supplementary material for: Double negative T cells decline in inflammatory reproductive dysfunction
Source: Sci Rep. 2026 Jun 21;16:28406. doi: 10.1038/s41598-026-57959-4 (PMC13562697; doi:10.1038/s41598-026-57959-4)
Supplement: Supplementary file 1 [file 41598_2026_57959_MOESM1_ESM.docx]

**Supplemental Data**

**Double negative T cells decline in inflammatory reproductive dysfunction**

Enitome E. Bafor^a,b*^, Toni Martin^a^, Bruna Karoline Tatematsu^c^, Ian A. Bettencourt^a^, Adrienne E. Kimmel^a^, Dorothy K. Sojka^c^, Howard A. Young^a^

^a^Cancer Innovation Laboratory, Center for Cancer Research, National Cancer Institute, Frederick, MD 21702, United States

^b^Present address: Department of Infectious Diseases and Immunology, College of Veterinary Medicine, University of Florida, Gainesville, Fl 32608, United States

^c^Microbiology and Immunology Department, Loyola University Health Science Campus, Maywood, IL 60153, United States

*Correspondence: bafor.enitome@ufl.edu

**1.0 Supplementary Materials and Method**

**1.1 Mouse genotyping**

Mice were genotyped at 3-4 weeks of age. When necessary, additional tail biopsies were collected from experimental animals at the time of sacrifice to confirm genotype. Genomic DNA was isolated from 2-3 mm tail clips by alkaline lysis. Briefly, 75 µl of alkaline lysis buffer (0.25 M NaOH, 5 mM EDTA, pH ~12) was added to each tube containing a tail piece and samples were incubated at 95°C for 60 min. After lysis, 75 µl of neutralization buffer (Tris-HCl, pH ~5) was added, tubes were vortexed to mix, and lysates were either used immediately for PCR or stored at -20°C until use. ARE-Del mice (ARE^-/-^ and ARE^+/-^ )were genotyped as previously described [1] using a three-primer PCR strategy with the following primers: WT ARE reverse, 5′-ATTTAAAAATTCAAATAGTGCTGGC-3′; common ARE forward, 5′-GTCAACAACCCACAGGTCCA-3′; and ARE^-/-^ reverse, 5′-CCGCGGTGGTACCATAACTT-3′. PCR reactions (20 µl) contained 2 µl tail DNA, 0.4 µl of 10 µM primer mix (combined forward and reverse primers), 10 µl 2x Platinum II Hot-Start Green PCR Master Mix (Invitrogen, Cat. 14001013), and 7.6 µl nuclease-free water. PCR was performed with an initial denaturation at 94°C for 2 min, followed by 35 cycles of 94°C for 15 s, 60°C for 15 s, and 68°C for 15 s, and a final hold at 4°C. Amplified products were resolved on 1.5% agarose gels prepared in 1x TBE containing GelRed and run at 100 V for approximately 1 h before imaging under UV illumination to assign genotypes.

**1.2 Estrous cycle**

The murine estrous cycle consists of four sequential stages, proestrus, estrus, metestrus, and diestrus, which differ in hormonal milieu and vaginal cytology. Proestrus corresponds to a follicular-like phase with rising estradiol and progesterone. Estrus encompasses ovulation and is characterized by high progesterone. Metestrus reflects early luteal activity. Diestrus is the longest phase, marked by relatively low circulating hormone levels and gradual re-accumulation of estradiol. Vaginal cytology was assessed on methylene blue–stained smears as described in the main Methods and stages were assigned according to established criteria [2-6]. Proestrus was identified by a predominance of nucleated epithelial cells with round to oval nuclei, with intermixed cornified epithelial cells and scattered leukocytes sometimes present. Estrus was defined by a dominance of anucleated, cornified squamous epithelial cells, often in clusters or sheets, with few or no leukocytes. This stage was used for most steady-state analyses of reproductive tissues in the main figures. Metestrus was characterized by a mixed population of leukocytes, nucleated epithelial cells, and cornified epithelial cells without a clearly dominant cell type, consistent with a transition from estrus to diestrus. Diestrus was identified by a predominance of leukocytes with fewer epithelial cells and represents the longest, hormonally quiescent phase of the cycle.

For synchronization, females were exposed to male-scented bedding for 48 h before experiments. Vaginal lavage was performed on the day of tissue collection and, where indicated, at the end of experiments to confirm cycle stage. Staging was performed by two independent observers, and only mice with concordant estrus cycles were included in estrus-restricted analyses.

**Table S1. Antibodies used for flow cytometry and cell sorting**

| Antibody | Fluorophore | Clone | Company |
| --- | --- | --- | --- |
| CD45 | Alexa Fluor-700 | 30-F11 | BioLegend |
| CD45 | BUV563 | 30-F11 | BD Biosciences |
| CD45.1 | BV785 | A20 | BioLegend |
| CD45.2 | FITC | 104 | BioLegend |
| CD3 | PE/dazzle594 | 17A2 | BioLegend |
| CD3 | PE | 17A2 | BD Pharmigen |
| CD4 | APC-eFluor780 | RM4-5 | Invitrogen, ThermoFisher Scientific |
| CD8a | FITC | 53-6.7 | BioLegend |
| CD8a | APC | 53-6.7 | BioLegend |
| CD8a | PE-Cy7 | 53-6.7 | BioLegend |
| NK1.1 | Brilliant Violet 711 | PK136 | BioLegend |
| TCRβ | APC | H57-97 | BioLegend |
| CD5 | BV421 | 53-7.3 | BioLegend |
| CD178 | PE | MFL3 | BioLegend |
| CD44 | PE | IM7 | BioLegend |
| CD44 | BUV395 | IM7 | BD Biosciences |
| CD62L | PerCPCy5.5 | MEL-14 | BioLegend |
| CD62L | PE-Cy5 | MEL-14 | BioLegend |
| Zombie | Aqua | - | BioLegend |
| S1PR1 (S1P1) | Alexa Fluor-405 | # 713412 | R&D systems |
| Ccr7 | BUV805 | 4B12 | BD Biosciences |
| CD69 | BUV661 | H1.2F3 | BD Biosciences |
| CD103 | BUV615 | M290 | BD Biosciences |
| CD25 | PE | PC61 | BD Biosciences |
| Runx3 | BV421 | R3-5G4 | BD Biosciences |

**Supplementary Results**

**Figure S1. Extended phenotypic profiling and transcriptional pathway enrichment in DNTs and CD8^+^ T cells across lymphoid and reproductive tissues.** (**A**) Representative gating strategy. Lymphocytes were gated by FSC-A vs SSC-A, followed by singlets (FSC-A vs FSC-H) and live cells (Zombie Aqua^-^). CD45^+^CD3^+^ cells were then gated, followed by TCRβ^+^ cells. CD4^+^ and CD8^+^ subsets were identified, and DNTs were defined as CD3^+^TCRβ^+^CD4^-^CD8^-^NK1.1^-^ cells. (**B-C**) Representative flow plots showing CD44 (**B**) and CD25 (**C**) staining on DNTs and CD8^+^ T cells in thymus, spleen, ovary, and uterus. Numbers indicate the percentage of CD44^+^ or CD25^+^ cells within each gate. (**D-E**) Frequency of CD44^+^ (**D**) and CD25^+^ (**E**) cells among thymic DNTs and CD8^+^ T cells. (n = 5-6 mice per group). (**F-G**) Representative overlaid histograms of TCRβ (**F**) and CD5 (**G**) expression in thymic CD8^+^ T cells (black line) and DNTs (blue shaded), normalized to mode. (**H**) Frequency of CD5^+^ and TCRβ^+^ cells within thymic DNT and CD8^+^ T cell gates (n = 3 mice per group). (**I-J**) Representative overlaid histograms of TCRβ expression in splenic, ovarian, and uterine CD8^+^ T cells (black line) and DNTs (blue shaded) (**I**), and CD5 expression in splenic, ovarian, and uterine CD8^+^ T cells and DNTs (**J**), normalized to mode. (**K-L**) Runx3 expression in DNTs and CD8^+^ T cells: frequency of Runx3^+^ cells in splenic and thymic DNTs and CD8^+^ T cells (**K**), including double-positive (DP; CD4^+^CD8^+^) thymocytes, and frequency of Runx3^+^ cells in ovarian and uterine DNTs and CD8^+^ T cells (**L**). (n = 3-5 mice per group). (**M**) T-bet expression in DNTs and CD8^+^ T cells from spleen, ovary, and uterus, shown as the percentage of T-bet^+^ cells in each subset (n = 7 mice per group). **(N-O)** Gene Ontology (GO) biological process (**N**) and KEGG pathway [7-9] (**O**) enrichment analyses of genes downregulated in SPDNT relative to SPCD8 (SPDNT-downregulated; n = 3 biological replicates per group). KEGG pathway enrichment was performed using KEGG pathway gene sets; no KEGG pathway map images were reproduced. Dot size reflects the number of genes in each term and color indicates adjusted p-value. Data in bar graphs are presented as mean ± SEM. Statistical comparisons include Mann-Whitney tests unless otherwise indicated; *p < 0.05, **p < 0.01, ***p < 0.001; ns, not significant.

**Figure S2. Gating strategy for identification and phenotypic analysis of CD8⁺ T cells and DNTs.** Representative flow cytometry gating used in this study (Example shown: ovary). From left to right, top row: events were displayed on FSC-A versus SSC-A to exclude debris, then gated on singlets (FSC-A versus FSC-H), and on live cells by exclusion of Zombie Aqua^+^ events. Middle row: within live cells, leukocytes were gated as CD45^+^, followed by T cells as CD45^+^CD3^+^ and further as CD3⁺TCRβ⁺. CD4 and CD8α expression was then used to define CD8^+^ T cells (CD3^+^TCRβ^+^CD8α^+^CD4^-^) and to exclude CD4^+^ T cells. Bottom row: DNTs were identified as CD3^+^TCRβ^+^CD4^-^CD8α^-^NK1.1^-^ cells. Within the CD8^+^ T-cell gate, CD44 and CD62L expression was used to distinguish naive (CD44^lo^CD62L^hi^) and effector-like (CD44^hi^CD62L^lo^) CD8^+^ T cells. Expression of residency-associated markers CD69 and CD103 and TCR-modulatory marker CD5 was assessed within the gated CD8^+^ T-cell and DNT subsets, as indicated. Plots are representative of gating strategies across multiple experiments. This figure shows the analytical flow cytometry gating strategy used for phenotypic analyses and for sorting in functional assays and adoptive transfer experiments; the RNA-seq sort used a reduced panel as described in methods section on RNA sequencing and bioinformatic analysis.

**Figure S3.** **DNTs do not suppress CD8^+^ T-cell proliferation across a transwell barrier.** (**A**) Schematic of the transwell coculture assay. CFSE-labeled splenic CD8^+^ T cells were plated in the lower chamber and stimulated with anti-CD3/CD28, while splenic DNTs were added to the upper transwell insert, physically separated from CD8^+^ T cells but sharing media. (**B**) Representative CFSE histograms of CD8^+^ T cells cultured unstimulated (Unstim), stimulated without DNTs (1:0), or stimulated with DNTs at the indicated CD8^+^ T cell:DNT ratios (2:1, 1:1) in the transwell system. (**C**) Quantification of suppression across the transwell barrier, calculated using percent divided cells and expressed as percent suppression relative to stimulated CD8^+^ T cells cultured without DNTs (1:0) (n = 3 biological replicates). Transwell separation did not support detectable suppression under these conditions, consistent with a primarily contact-dependent mechanism under these conditions. Bars show mean ± SEM; statistical testing was done using Kruskal-Wallis test; ns, not significant.

**Figure S4**. Representative post-sort reanalysis of FACS-sorted splenic DNTs used in functional assays. Cells were sorted as live CD45^+^CD3^+^TCRβ^+^CD4^-^CD8α^-^NK1.1^-^ and reanalyzed immediately after sorting to confirm purity. DNT-like purity: >90.0%; NK1.1- confirmation: >90.0%.

**Figure S5**. **Selected transcriptional features distinguishing splenic and thymic DNTs from CD8⁺ T cells.** Heatmap of selected genes associated with checkpoint/regulatory state, lineage identity, tissue residency/trafficking, and effector or survival programs across splenic DNTs (SPDNT), thymic DNTs (THDNT), splenic CD8^+^ T cells (SPCD8), and thymic CD8^+^ T cells (THCD8). Columns represent individual biological replicates (n = 3 per group), and rows represent the indicated genes. Expression values are row-scaled (z-scores), with warmer colors indicating relatively higher expression and cooler colors indicating relatively lower expression within each gene across samples. This panel is shown as supplementary support for the transcriptional distinction between DNT and CD8^+^ T-cell populations. Gene-level differential expression statistics for these markers are provided in Table S8.

**Figure S6. CD8^+^ T-cell depletion alters DNT numbers in the uterus and lymphoid tissues.**
DNT numbers in uterus (**A**), lymph nodes (**B**), and spleen (**C**) from cycling WT females treated with depleting anti-CD8α mAb (a-CD8) or isotype IgG control, as described in Fig. 3C. DNTs were defined as live CD45^+^CD3^+^TCRβ^+^CD4^-^CD8^-^NK1.1^-^ cells. Data shown are from one representative experiment (n = 3 mice per group); the experiment was repeated independently. Bars show mean ± SEM of absolute DNT counts per tissue with individual mice overlaid. Statistical comparisons were performed using unpaired two-tailed t tests; *p < 0.05.

**Figure S7. Residency-associated marker expression and parabiosis chimerism features of DNTs compared with CD8^+^ T cells. (A)** Representative flow cytometry plots showing CD69 and CD103 expression on CD8^⁺^ T cells (left) and DNTs (right) from ovary and uterus of WT females. Numbers indicate the percentage of cells in each quadrant. **(B)** Representative plots showing S1PR1 and CCR7 expression on CD8^+^ T cells and DNTs from ovary and uterus. **(C)** S1PR1 and CCR7 expression on CD8^+^ T cells and DNTs from blood and lymph nodes (LN), illustrating enrichment of S1PR1^-^CCR7^-^ cells in reproductive tissues relative to circulating and lymphoid compartments. **(D-E)** Host-derived and partner-derived chimerism within DNT **(D)** and CD8^+^ T cell **(E)** gates in blood, spleen, and LN from CD45.1/CD45.2 parabiotic pairs at 5 weeks post-surgery. Symbols denote subsets as follows: host-derived circulating DNTs (cHost DNTs, open circles), partner-derived circulating DNTs (cDonor DNTs, open squares), host-derived circulating CD8^+^ T cells (cHost CD8Ts, open diamonds), partner-derived circulating CD8^+^ T cells (cDonor CD8Ts, open triangles), and CD69^+^ and/or CD103^+^ TRM-like subsets (filled circles; shown in blue for DNT TRM in panel D and in black for CD8^+^ TRM in panel E). Data are expressed as the percentage of total DNTs **(D)** or total CD8^+^ T cells **(E)** within each tissue. **(F-G)** Representative CD45.1 versus CD45.2 plots showing host- and partner-derived DNTs (gated as CD3^+^NK1.1^-^CD4^-^CD8^-^) from ovary and uterus at 2 weeks **(F)** and 5 weeks **(G)** post-parabiosis in CD45.1 parabionts. Parabiosis data are from two independent cohorts with n = 5 parabiotic pairs per cohort. Bars show mean ± SEM; individual mice are overlaid. Statistical comparisons were performed using two-way ANOVA with Tukey’s multiple-comparisons test; *p < 0.05, **p < 0.01, ***p < 0.001, ****p < 0.0001.

**Figure S8**. **DNT and CD8^+^ T-cell dynamics in blood and reproductive tissues in ARE^-/-^ females and during antigen-driven ZP3-CFA immunization. (A)** Frequencies of DNTs among total CD3^+^ T cells in blood, spleen, and lymph nodes (LN) from nonpregnant (NP) WT, ARE^+/-^, and ARE^-/-^ females (n = 4-10 mice per group). (**B-C**) Representative flow cytometry plots showing CD8^+^ T cells (top row) and DNTs (bottom row) in ovary (**B**) and uterus (**C**) from NP WT, ARE^+/-^, and ARE^-/-^ females, gated on CD3^+^ T cells and excluding NK1.1^+^ events. Numbers indicate the percentage of cells in each gate. (**D**) Representative plots of CD8^+^ T cells and DNTs in the ovary of ZP3 peptide-immunized (ZP3^+^) and unimmunized (ZP3^-^) WT females, showing CD8^+^ T cell expansion and reduced DNT frequency following antigen-specific ovarian inflammation. (**E-F**) Distribution of CD8^+^ T-cell subsets in the ovary (**E**) and uterus (**F**) at 6.5 days post coitum (dpc) in WT, ARE^+/-^, and ARE^-/-^ females (n = 3-4 mice per group). CD8^+^ T-cell subsets were defined by CD44 and CD62L as effector/effector-memory (T_E/EM_; CD44^hi^CD62L^lo^), central memory (T_CM_; CD44^hi^CD62L^hi^), and naive (T_N_; CD44^lo^CD62L^hi^). Data are shown as the percentage of total CD8^+^ T cells in each subset. (**G**) Representative CD44 versus CD62L plots showing T_E/EM_, T_CM_, and T_N_ distributions in ovary and uterus at 6.5 dpc across WT, ARE^+/-^, and ARE^-/-^ females. Bar graphs show mean ± SEM with individual mice indicated. Statistical comparisons across genotypes were performed using Two-way ANOVA with Tukey’s multiple comparison test; *p < 0.05, **p < 0.01, ****p < 0.0001.

**Tables S2-S5 are available as excel file attachments.**

**Table S6. Differential expression of effector and activation genes in splenic and thymic DNTs versus splenic and thymic CD8⁺ T cells (log₂FC and adjusted p-values)** SPDNT = splenic DNT; SPCD8 = splenic CD8^+^ T cells; THDNT = thymic DNTs; THCD8 = thymic CD8⁺ T cells.

| Gene | log_2_FC  SPDNT vs SPCD8 | p-value  SPDNT vs SPCD8 | Padj  SPDNT vs SPCD8 | log_2_FC  THDNT vs THCD8 | p-value  THDNT vs THCD8 | Padj  THDNT vs THCD8 |
| --- | --- | --- | --- | --- | --- | --- |
| Il10 | 5.025 | 0.0684 | 0.183 | 3.434 | 0.226 | 0.562 |
| Ifng | 2.131 | 0.0703 | 0.186 | 2.065 | 0.0814 | 0.294 |
| Cd44 | 1.209 | 2.37e-05 | 0.00027 | 0.486 | 0.0904 | 0.316 |
| Gzmb | -0.623 | 0.32 | 0.526 | 1.71 | 0.00648 | 0.0443 |
| Lag3 | 2.596 | 3.5e-17 | 2.19e-15 | 3.585 | 3.95e-33 | 8.56e-31 |
| Il2ra | 2.905 | 1.01e-23 | 1e-21 | 2.468 | 2.17e-19 | 2e-17 |
| Tigit | 1.792 | 0.0631 | 0.172 | 2.798 | 0.0031 | 0.0245 |
| Pdcd1 | 4.271 | 1.61e-29 | 2.33e-27 | 4.992 | 3.93e-54 | 2.21e-51 |
| Cxcr3 | 0.024 | 0.958 | 0.987 | 0.473 | 0.315 | 0.671 |
| Tbx21 | 0.923 | 0.00165 | 0.01 | 2.395 | 3.55e-12 | 1.63e-10 |
| Eomes | 1.005 | 0.000868 | 0.00591 | 0.273 | 0.376 | 0.729 |
| Sell | -0.783 | 0.00927 | 0.04 | -0.009 | 0.977 | 1 |
| Prf1 | -1.626 | 3.99e-16 | 2.23e-14 | -1.79 | 3.91e-18 | 3.26e-16 |
| S1pr1 | -0.459 | 0.0739 | 0.193 | -1.321 | 2.85e-07 | 6.48e-06 |
| Trbc2 | -1.447 | 1.87e-10 | 5.66e-09 | -0.633 | 0.00527 | 0.0376 |
| Trbc1 | -1.306 | 1.95e-08 | 4.44e-07 | -0.2 | 0.388 | 0.739 |
| Cd5 | -0.984 | 2.55e-05 | 0.00028 | -0.955 | 4.13e-05 | 0.000596 |
| Trac | -2.301 | 2.5e-21 | 2.13e-19 | -2.058 | 1.91e-17 | 1.49e-15 |
| Cd8a | -7.277 | 2.63e-71 | 3.26e-68 | -7.643 | 2.17e-78 | 2.49e-75 |
| Batf | -0.915 | 0.0219 | 0.0777 | 0.658 | 0.097 | 0.332 |
| Runx3 | -0.185 | 0.255 | 0.455 | 0.279 | 0.0847 | 0.302 |

**Table S7. Differential expression of exhaustion and regulatory genes for Splenic WT DNT and CD8^+^T cells genes (log₂FC and adjusted p values)** SPDNT = splenic DNT; SPCD8 = splenic CD8^+^T cells.

| Gene | Ensembl ID | log_2_FC SPDNT vs SPCD8 | padj SPDNT vs SPCD8 |
| --- | --- | --- | --- |
| Tnf | ENSMUSG00000024401.14 | -0.660378652 | 0.49506938 |
| Prf1 | ENSMUSG00000037202.6 | -1.626470404 | 1.63e-28 |
| Foxp3 | ENSMUSG00000039521.13 | -0.599843922 | 0.55332001 |
| Tox | ENSMUSG00000041272.11 | 0.685058079 | 0.001143886 |
| Rag1 | ENSMUSG00000061311.6 | 6.264327941 | 0.000148613 |
| Gzmb | ENSMUSG00000015437.5 | -0.624414504 | 0.521680283 |
| Pdcd1 | ENSMUSG00000026285.7 | 4.270338672 | 1.10e-38 |
| Ctla4 | ENSMUSG00000026011.13 | 0.982093472 | 0.546108643 |
| Lag3 | ENSMUSG00000030124.2 | 2.594115677 | 6.14e-29 |
| Ifng | ENSMUSG00000055170.4 | 2.13030141 | 0.177810939 |
| Il10 | ENSMUSG00000016529.5 | 5.023224351 | 0.209409956 |

**Table S8.** **Differential expression of tissue-residency and checkpoint genes in splenic and thymic DNTs versus splenic and thymic CD8⁺ T cells (log₂FC and adjusted p-values)** SPDNT = splenic DNT; SPCD8 = splenic CD8^+^ T cells; THDNT = thymic DNTs; THCD8 = thymic CD8⁺ T cells.

| Gene | log_2_FC  SPDNT vs SPCD8 | p-value SPDNT vs SPCD8 | Padj SPDNT vs SPCD8 | log_2_FC THDNT vs THCD8 | p-value  THDNT vs THCD8 | Padj THDNT vs THCD8 |
| --- | --- | --- | --- | --- | --- | --- |
| Gzmb | -0.623 | 0.32 | 0.526 | 1.71 | 0.00648 | 0.0443 |
| Lag3 | 2.596 | 3.5e-17 | 2.19e-15 | 3.585 | 3.95e-33 | 8.56e-31 |
| Il2ra | 2.905 | 1.01e-23 | 1e-21 | 2.468 | 2.17e-19 | 2e-17 |
| Tigit | 1.792 | 0.0631 | 0.172 | 2.798 | 0.0031 | 0.0245 |
| Pdcd1 | 4.271 | 1.61e-29 | 2.33e-27 | 4.992 | 3.93e-54 | 2.21e-51 |
| Gata3 | 0.168 | 0.268 | 0.47 | 3.003 | 1.27e-89 | 2.24e-86 |
| Runx3 | -0.185 | 0.255 | 0.455 | 0.279 | 0.0847 | 0.302 |
| Foxp3 | -0.599 | 0.363 | 0.569 | -0.39 | 0.506 | 0.826 |
| Tox | 0.686 | 0.000327 | 0.0026 | 0.987 | 1.77e-07 | 4.2e-06 |
| Rag2 | 2.843 | 0.146 | 0.312 | 0.149 | 0.913 | 1 |
| Rag1 | 6.269 | 8.58e-06 | 0.000107 | 0.233 | 0.75 | 0.955 |
| Cd8b1 | -5.925 | 6.68e-139 | 1.24e-134 | -6.793 | 7.03e-171 | 1.62e-166 |
| Cd8a | -7.277 | 2.63e-71 | 3.26e-68 | -7.643 | 2.17e-78 | 2.49e-75 |
| Cd5 | -0.984 | 2.55e-05 | 0.000283 | -0.955 | 4.13e-05 | 0.000596 |
| Itgae | -2.092 | 5.49e-24 | 5.46e-22 | -2.476 | 4.35e-34 | 1.01e-31 |
| Ctla4 | 0.983 | 0.353 | 0.559 | -2.865 | 0.00683 | 0.0462 |
| Bcl2 | -0.023 | 0.92 | 0.968 | -0.277 | 0.222 | 0.557 |
| Ifng | 2.131 | 0.0703 | 0.186 | 2.065 | 0.0814 | 0.294 |
| Tbx21 | 0.923 | 0.00165 | 0.01 | 2.395 | 3.55e-12 | 1.63e-10 |
| Eomes | 1.005 | 0.000868 | 0.00591 | 0.273 | 0.376 | 0.729 |
| Cxcr6 | -0.974 | 0.00415 | 0.021 | -0.448 | 0.189 | 0.508 |
| Il7r | -1.155 | 2.27e-08 | 5.09e-07 | -0.42 | 0.0426 | 0.187 |
| Prf1 | -1.626 | 3.99e-16 | 2.23e-14 | -1.79 | 3.91e-18 | 3.26e-16 |
| Cd69 | 0.68 | 0.461 | 0.659 | -0.738 | 0.424 | 0.764 |
| S1pr1 | -0.459 | 0.0739 | 0.193 | -1.321 | 2.85e-07 | 6.48e-06 |
| Foxo1 | 0.13 | 0.45 | 0.651 | -0.362 | 0.035 | 0.161 |

**Supplementary References**

[1]	D. L. Hodge, C. Berthet, V. Coppola, W. Kastenmüller, M. D. Buschman, P. M. Schaughency *et al.* IFN-gamma AU-rich element removal promotes chronic IFN-gamma expression and autoimmunity in mice, Journal of autoimmunity.53 (2014) 33–45.

[2]	G. A. Wood, J. E. Fata, K. L. Watson, R. Khokha. Circulating hormones and estrous stage predict cellular and stromal remodeling in murine uterus, Reproduction.133 (2007) 1035–1044.

[3]	M. C. Cora, L. Kooistra, G. Travlos. Vaginal Cytology of the Laboratory Rat and Mouse: Review and Criteria for the Staging of the Estrous Cycle Using Stained Vaginal Smears, Toxicol Pathol.43 (2015) 776–793.

[4]	C. H. Hubscher, D. L. Brooks, J. R. Johnson. A quantitative method for assessing stages of the rat estrous cycle, Biotech Histochem.80 (2005) 79–87.

[5]	K. Bertolin, B. D. Murphy. 7 - Reproductive Tract Changes During the Mouse Estrous Cycle. In: Croy BA, Yamada AT, DeMayo FJ, Adamson SL, editors. The Guide to Investigation of Mouse Pregnancy, Boston: Academic Press; 2014, p. 85–94.

[6]	E. E. Bafor, F. Ukpebor, U. Elvis-Offiah, A. Uchendu, O. Omoruyi, G. U. Omogiade. Justicia flava Leaves Exert Mild Estrogenic Activity in Mouse Models of Uterotrophic and Reproductive Cycle Investigations, J Med Food.23 (2020) 395–408.

[7]	M. Kanehisa. Toward understanding the origin and evolution of cellular organisms, Protein Sci.28 (2019) 1947–1951.

[8]	M. Kanehisa, M. Furumichi, Y. Sato, Y. Matsuura, M. Ishiguro-Watanabe. KEGG: biological systems database as a model of the real world, Nucleic Acids Res.53 (2025) D672–d677.

[9]	M. Kanehisa, S. Goto. KEGG: kyoto encyclopedia of genes and genomes, Nucleic Acids Res.28 (2000) 27–30.
